# Identification of a poly-cyclopropylglycine–containing peptide *via* bioinformatic mapping of radical S-adenosylmethionine enzymes

**DOI:** 10.1016/j.jbc.2022.101881

**Published:** 2022-03-31

**Authors:** Anastasiia Kostenko, Yi Lien, Aigera Mendauletova, Thacien Ngendahimana, Ivan M. Novitskiy, Sandra S. Eaton, John A. Latham

**Affiliations:** Department of Chemistry and Biochemistry, University of Denver, Denver, Colorado, USA

**Keywords:** radical-S-adenosylmethionine enzyme, sequence similarity network, ribosomally synthesized and posttranslationally modified peptide, bioinformatics, TVG biosynthesis, BGC, biosynthetic gene cluster, CPG, cyclopropylglycine, DMSO, dimethylsulfoxide, EFI-EST, Enzyme Function Initiative Enzyme Similarity Tool, EFI-GNT, EFI’s genome neighborhood tool, EPR, electron paramagnetic resonance, HSQC, heteronuclear single quantum coherence, MS, mass spectrometry, RiPP, ribosomally synthesized and post-translationally modified peptide, rSAM, radical-SAM, SSN, sequence similarity network

## Abstract

Peptide-derived natural products are a large class of bioactive molecules that often contain chemically challenging modifications. In the biosynthesis of ribosomally synthesized and posttranslationally modified peptides (RiPPs), radical-SAM (rSAM) enzymes have been shown to catalyze the formation of ether, thioether, and carbon-carbon bonds on the precursor peptide. The installation of these bonds typically establishes the skeleton of the mature RiPP. To facilitate the search for unexplored rSAM-dependent RiPPs for the community, we employed a bioinformatic strategy to screen a subfamily of peptide-modifying rSAM enzymes which are known to bind up to three [4Fe-4S] clusters. A sequence similarity network was used to partition related families of rSAM enzymes into >250 clusters. Using representative sequences, genome neighborhood diagrams were generated using the Genome Neighborhood Tool. Manual inspection of bacterial genomes yielded numerous putative rSAM-dependent RiPP pathways with unique features. From this analysis, we identified and experimentally characterized the rSAM enzyme, TvgB, from the *tvg* gene cluster from *Halomonas anticariensis*. In the *tvg* gene cluster, the precursor peptide, TvgA, is comprised of a repeating TVGG motif. Structural characterization of the TvgB product revealed the repeated formation of cyclopropylglycine, where a new bond is formed between the γ-carbons on the precursor valine. This novel RiPP modification broadens the functional potential of rSAM enzymes and validates the proposed bioinformatic approach as a practical broad search tool for the discovery of new RiPP topologies.

Ribosomally synthesized and posttranslationally modified peptides (RiPPs) are a large and diverse family of natural products (1). RiPPs have gained attention as a potential source of biologically active molecules (2) and because they serve important physiological roles in host species (3, 4, 5). In general, RiPP biosynthesis is carried out by the extensive chemical modification of a genetically encoded precursor peptide. Following synthesis of the peptide by the ribosome, diverse families of tailoring enzymes catalyze chemical transformations on the peptide. Radical-SAM (rSAM) enzymes have emerged as a common tailoring enzyme in RiPPs biosynthesis. Characterization of rSAM enzymes associated with RiPP pathways have revealed extraordinary chemical approaches to modify peptides into complex natural products and have led to the discovery of unprecedented RiPP classes and novel antibiotics (6). To achieve their complex modifications, rSAM enzymes employ iron-sulfur clusters to reductively cleave SAM, generating a 5′-deoxyadenosyl radical (Fig. 1*A*). The highly reactive radical abstracts a hydrogen atom from the peptide substrate leading to various outcomes. Some outcomes include the formation of carbon-carbon bonds (7, 8, 9), carbon-sulfur bonds (10, 11, 12, 13, 14, 15), carbon-oxygen bonds (16), epimerization (17, 18), and methyltransfers (19, 20, 21), among other unique transformations (22), on the precursor peptide (Fig. 1*B*). The repertoire of natural products that depend on rSAM enzymes for critical modifications is growing and currently includes antibiotics (*e.g.*, (6, 23)), essential redox cofactors (*e.g.*, (9, 24)), and quorum-sensing molecules (*e.g.*, (16, 25)). Here, we describe a subset of RiPPs that require rSAM enzymes to install critical structural conformations on the precursor peptides, which ultimately serve as the skeleton for the mature RiPP. We refer to these as rSAM-dependent RiPPs.

**Figure 1:**
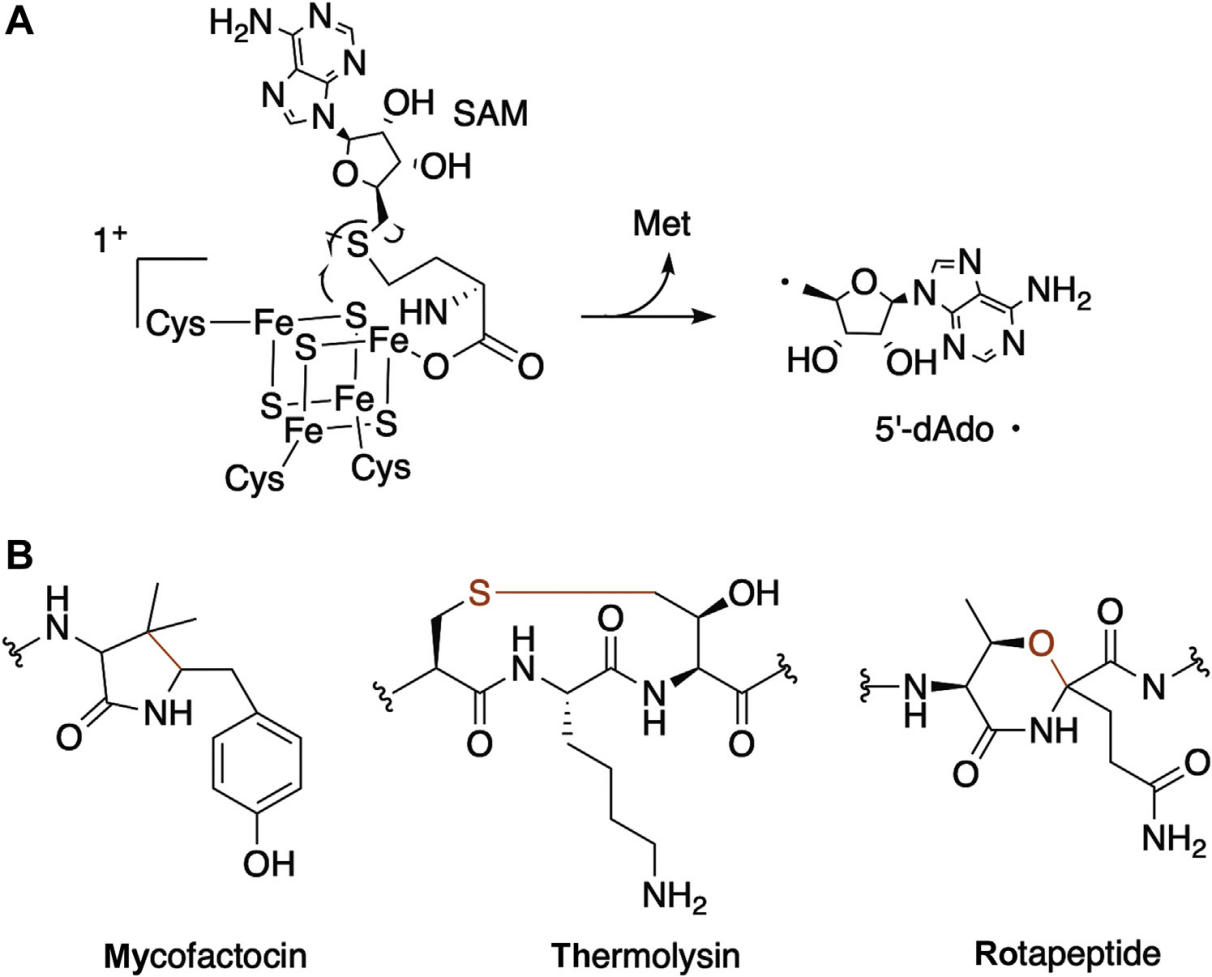
**Radical S-adenosylmethionine chemistry and possible outcomes.***A*, a representative reaction scheme depicting the homolytic cleavage of S-adenosylmethionine (SAM) to form a 5′-deoxyadenosyl radical (5′-dAdo⋅) by rSAM enzymes. *B*, some outcomes of rSAM enzyme–catalyzed modification of peptides include the formation of carbon-carbon bonds (*e.g.*, mycofactocin), carbon-sulfur bonds (*e.g.*, thermolysin), and carbon-oxygen bonds (*e.g.*, rotapeptide). rSAM, radical-SAM.

Most rSAM-dependent RiPPs discovered to date can be traced back to a bioinformatic analysis conducted by Haft and Basu in 2011 (10). In this study, Haft and Basu used the association of a subfamily of rSAM proteins as molecular markers, to discover new RiPP biosynthetic pathways (10). The subfamily that was examined consisted of rSAM proteins with an elongated C-terminal domain that binds one or two additional iron-sulfur clusters (26). This extra domain is annotated as SPASM named after the products synthesized by founding enzymes (subtilosin A, pyrroloquinoline quinone, anaerobic sulfatase, and mycofactocin). Haft and Basu utilized partial phylogenetic profiling and hidden Markov models to identify rSAM-SPASM proteins that were genetically clustered with putative precursor peptides. As a result, five RiPP natural product families and dozens of unique rSAM-SPASM subfamilies were identified. Since their initial study, approximately ten identified rSAM-SPASM proteins have been shown to be critical for the maturation of RiPP natural products (*e.g.*, (13, 27, 28)) and it is likely that many more will be validated.

It has been 10 years since the pivotal Haft and Basu study. Considering this, we sought to expand the bioinformatic analysis of rSAM-SPASM proteins to aid in identifying potentially new classes of rSAM-dependent RiPPs. In this study, we generated a sequence similarity network (SSN) of the IPR023867 subfamily of rSAM enzymes, which include rSAM-SPASM proteins. From this SSN, we used the genes in the first 118 clusters to generate gene neighborhood networks to identify putative RiPP pathways. These files will be made available as Supporting Information for anyone to use as a starting point. Among the identified rSAM-dependent RiPPs pathways, we characterized an rSAM-SPASM enzyme from a novel pathway, to validate our approach, which we named TVG. In TVG biosynthesis, the presumptive peptide, TvgA, has highly repetitive structure, suggesting that the same modification occurs multiple times. Indeed, we provide compelling evidence that the bioinformatically identified rSAM enzyme, TvgB, installs the same modification on TvgA multiples times, resulting in an unprecedented RiPP topology. We structurally characterize the modification using high-resolution-mass spectrometry and NMR spectroscopy. Overall, relying on data presented here, the proposed bioinformatic analysis proved itself as an effective search tool for the new RiPP structural topology.

## Results

### Discovery of the RiPP biosynthetic pathways

Since rSAM enzymes are strongly associated to RiPP biosynthesis, we expected that the relationship could be leveraged to discover new rSAM chemistries and interesting RiPP natural products. In particular, the IPR023867 rSAM subfamily consists of ∼24,000 protein sequences (at the time of analysis) including many rSAM enzymes that are known (13) or expected (10) to modify peptides. To identify new RiPP natural products, the Enzyme Function Initiative Enzyme Similarity Tool (EFI-EST) (29) was used to create a SSN for the IPR023867 subfamily. In short, UniRef90 sequences of the IPR023867 protein family (∼11,000 sequences) were used in the initial SSN analysis. Of the UniRef90 sequences, the vast majority (91%) belong to the Bacteria superkingdom with Archaea (6.5%), Viruses (<1%), and Eukaryota (<1%) superkingdoms also being represented. Within the Bacteria superkingdom, UniRe90 sequences were primarily from Proteobacteria (∼27%), Firmicutes (∼12%), Actinobacteria (∼11%), and the Bacteriodetes (∼8%) phyla.

**Figure 2:**
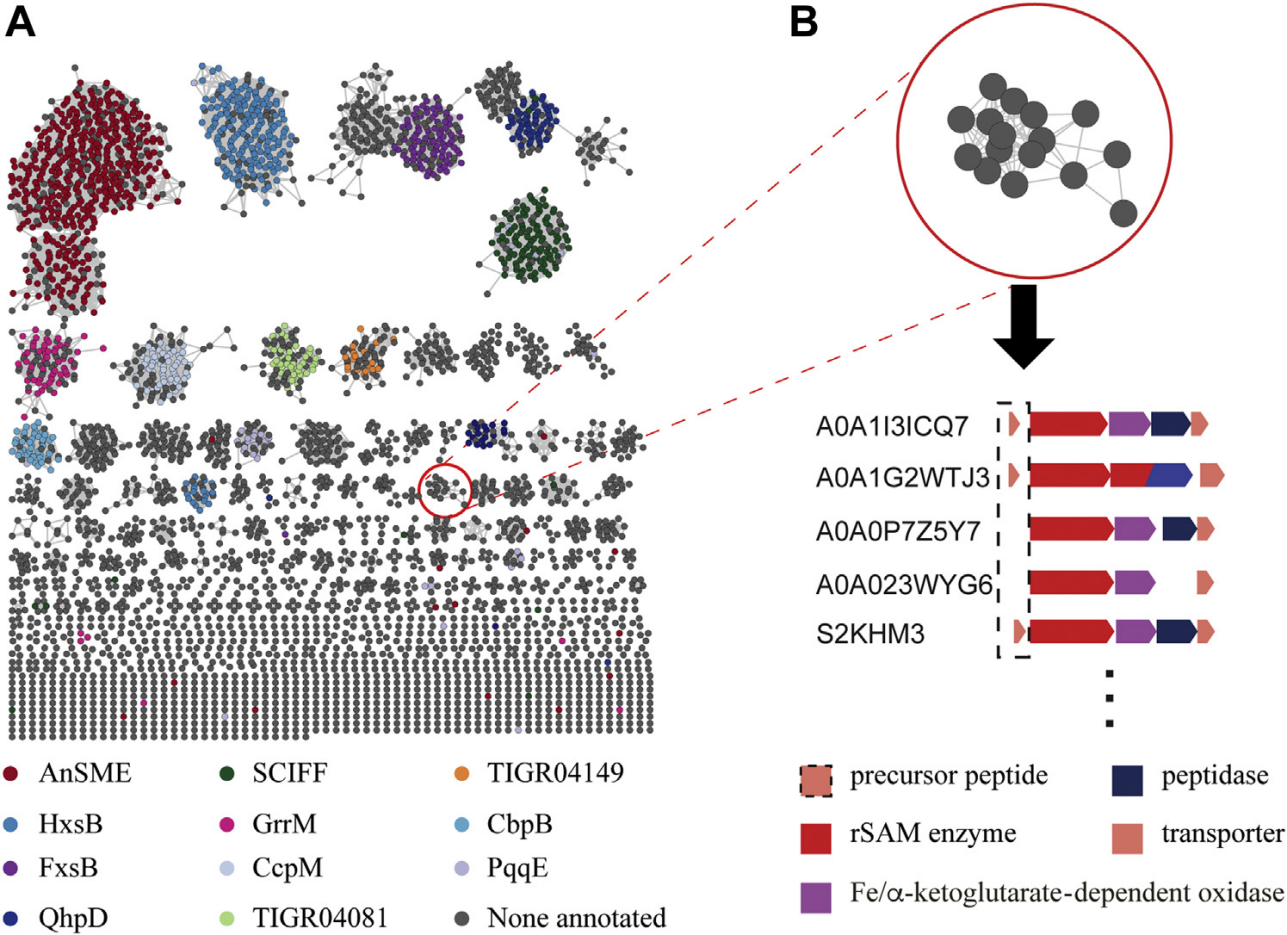
**A bioinformatic workflow for rSAM enzymes.***A*, the sequence similarity network of the IPR023867 family of rSAM enzymes using the Uniref90 database, an E value of 10^−80^, and a 40% sequence identify cutoff. Clusters were colored by gene description. *B*, the representative sequence for each cluster was used to generate a genome neighborhood diagram from which precursor peptides were identified and used in subsequent sequence alignments. rSAM, radical-SAM.

To finalize the SSN, an E-value of 10^−80^ and a sequence identity of 40% were used. This resulted in an SSN that contained >4000 nodes distributed in >250 clusters (Figs. 2*A* and S1). Lastly, clusters were colored by gene description to show that, for the most part, this analysis grouped known or expected enzymes. As shown in Figure 2*A*, many of the larger clusters are associated with known enzymes such as AnSME (30) and SCIFF (13), or previously annotated enzymes such as CcpM, and HxsB (10). However, these make up a small fraction of the total number of clusters represented.

**Figure 3:**
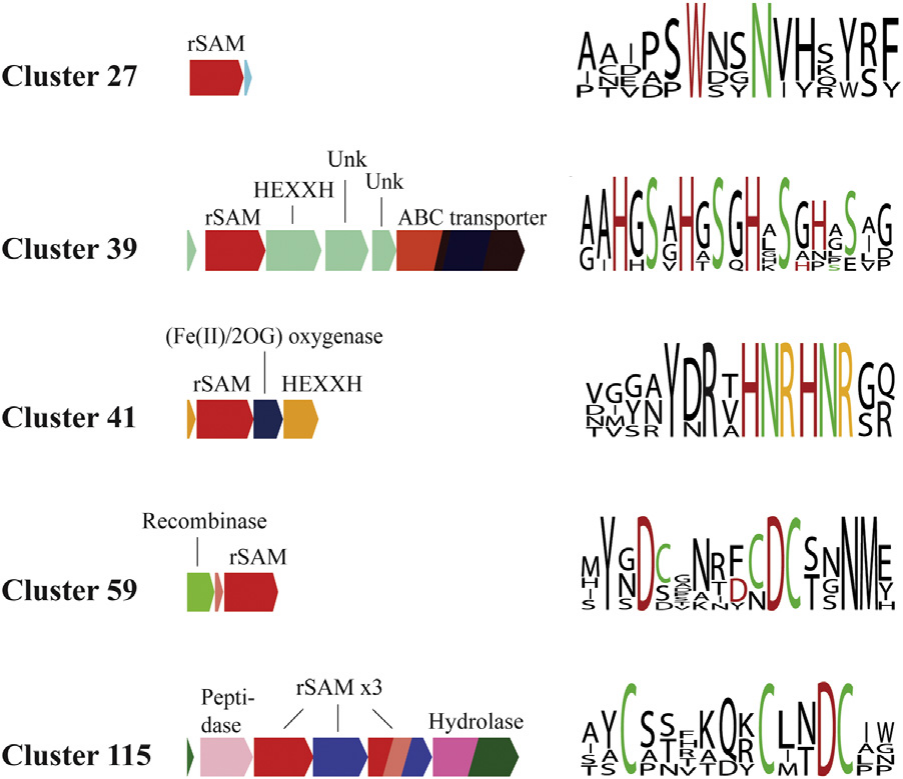
**Selected examples of bioinformatically identified RiPP biosynthetic pathways.** Using the clusters identified from a sequence similarity network analysis, the representative sequence from each node was used to generate a genome neighborhood diagram. From the genome neighborhood diagrams, precursor peptides were identified and aligned to detect core regions where the modifications are expected to occur. Conserved amino acid residues that presumably participate in the modification are highlighted in color. RiPP, ribosomally synthesized and post-translationally modified peptide.

Our subsequent analyses focused on the first 118 clusters since they contained multiple nodes and more than three sequences total. The gene context of each cluster in the resulting SSNs was analyzed to identify putative RiPP biosynthetic pathways. The EFI’s genome neighborhood tool (EFI-GNT) (31) was used to create genome neighborhood diagrams of representative rSAM sequences within each gene cluster (*e.g.*, Fig. 2*B*). Multiple annotated genomes represented within a cluster were searched to identify a putative peptide within the rSAM-associated gene cluster. If a peptide was annotated and/or other known modifying enzymes (*e.g.*, peptidase) were present, it was assumed to be a RiPP biosynthetic pathway. However, if a peptide was not annotated, a 1000 base pair region up and downstream of the gene cluster was analyzed by OrfFinder (32) to identify potential precursor peptides. Multiple peptides with sequence consensus were identified as putative RiPP biosynthetic precursors. Using this workflow, we annotated 22 putative biosynthetic pathways that have yet to be reported on (Figs. 3 and S2), some of which will be featured below. We have made the SSN file and the gene alignment files for each cluster available as Supporting Information.

Here, we highlight clusters where the putative peptide substrate was apparent, putative modification site(s) were well defined by sequence consensus, and the expected topologies of RiPP products are unique. To begin with, we examined Cluster 27 which contains 12 homologous rSAM enzymes. Upon examining the gene context of the rSAM enzymes, we found that ten homologs are colocalized with a single peptide (Fig. 3). The sequence alignment of the putative precursor peptide shows a conserved C-terminal region consisting of two repeating Aro-Xaa-Xaa-Asn motifs, where Aro is either the aromatic residues Trp or Tyr. This motif is reminiscent of the Aro-Xaa-Asn motif found in the strained cyclophane RiPPs (28) and suggests that RiPP products that are produced by Cluster 27 rSAM enzymes are similar. Because of this, we expect Cluster 27-associated RiPP products to consist of a new cyclophane topology where the products contain 16- and 17-membered rings rather than 13- and 14-membered rings associated with known cyclophanes (28).

A second unique RiPP biosynthetic pathway that we found relies on rSAM proteins located in Cluster 59 (Fig. 3). The biosynthetic gene cluster (BGC) consists of only three genes, a recombinase, a rSAM enzyme, and a peptide. Encoded in the peptide sequence is a conserved Asp-Cys motif, which depending on the organism, is repeated up to six times. Since there are no published examples of similar motifs, it is difficult to predict what modification is a result of the associated rSAM enzyme. However, the Asp-Cys motif did appear in a second biosynthetic pathway. The BGC associated with rSAM enzymes in Cluster 115 encodes for 3 to 5 rSAM enzymes, a peptidase, and a hydrolase (Fig. 3). The precursor peptide contains at least three conserved Cys residues, one being a part of the Asp-Cys motif found in Cluster 59. While it is difficult to know what the product of the RiPP pathway is, we expect that multiple intramolecular thioether bonds are installed by the associated rSAM enzymes and that at least one rSAM enzyme modifies the Asp-Cys motif like rSAM enzymes associated with Cluster 59.

Certainly, many of the obvious RiPP pathways that we found were akin to known pathways that contain two or three genes (*e.g.*, peptide, rSAM enzyme, and an optional transporter), mainly those whose products contain intramolecular thioether bonds (*e.g.*, sacti/ranthipeptides) or cyclophanes. However, our SSN/genome neighborhood analysis also yielded a significant portion of large BGCs, much like what was found to be associated with Cluster 115. For instance, Cluster 41 contains rSAM enzymes that belong to a four-component BGC (Fig. 3). The BGC associated with rSAM enzyme in Cluster 41 consists of an rSAM enzyme, a putative α-ketoglutarate/Fe-dependent oxidase, and a putative peptidase. Here, the precursor peptide contains a conserved and repeating Tyr-Asn-Arg-Xaa-(His-Asn-Arg)_2_ repeat. While there is a precedent for a crosslink between Arg and Tyr in the “RRR” system (33), the sequence and the repeating nature of the “H(Y)NR” peptide is distinct. Although it could be predicted that at least one cyclophane is present between Tyr and Arg, it is difficult to know if a similar crosslinking occurs with His and Arg. Moreover, the function of the Fe-dependent oxidase is currently unknown and complicates our ability to predict the outcome of the pathway. Likewise, it is unknown where the putative cleavage site for the putative peptidase is, which calls into question if the RiPP product is three similar units (YNR, HNR, HNR) or a single unit with three or more modifications.

A more extreme example of a large BGC that was found using our SSN/GNN analysis is associated with Cluster 3. Cluster 3 consists of ∼100 rSAM proteins that have diverse gene contexts with very little overlap. From this Cluster, we found a BGC in a single organism, *Mucilaginibacter lappiensis*, which consists of six precursor peptides, two rSAM enzymes, an alcohol dehydrogenase, two proteins with unknown function and no known close homologs, and a thiopeptide-like lantibiotic dehydratase (Fig. 3). Five of the precursor peptides are nearly identical and the sixth contains a conserved His-Xaa-Ser motif. This His-Xaa-Ser motif is similar to the one identified by Haft and Basu (10), which putatively rely on the rSAM enzymes HxsB and HxsC for maturation. However, the rSAM enzymes associated with this BGC are not related to HxsB and HxsC (Clusters 2 and 33, respectively), which could indicate that either the modification installed by the two rSAM enzymes are unique to this system or that the conservation of the His-Xaa-Ser motif is coincidental. Regardless, it is likely that the product of this BGC will have a topology that is different from currently known RiPPs and thus characterization will be a fruitful endeavor.

### Identification of the TVG biosynthetic pathway

**Figure 4:**
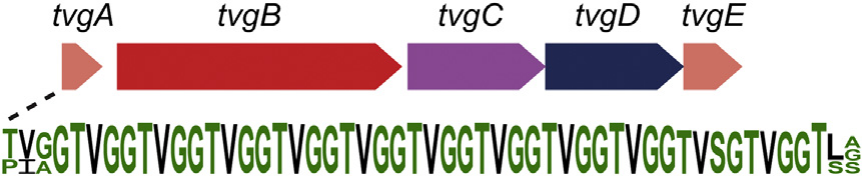
**A depiction of the TVG biosynthetic pathway which consists of the precursor peptide TvgA, the rSAM enzyme TvgB, the Fe/a-ketoglutarate dependent oxidase TvgC, and the peptidase/transporters TvgD and TvgE.** An alignment of TVG precursor peptides reveals a repeating TVGG motif. rSAM, radical-SAM.

To demonstrate that the described workflow can identify new rSAM-dependent RiPP natural products, we focused our efforts on the family of rSAM enzymes located in Cluster 40. This SSN cluster was chosen because (1) the precursor peptide had high sequence homology, (2) the precursor peptide contained a repeating core region which facilitated prediction of rSAM chemistry, and (3) proteins within the gene cluster are well conserved with minor variations. This latter point is demonstrated in the gene context alignment of Cluster 40, which suggests that at least two similar biosynthetic pathways are present (Fig. 2*B*). The most abundant BGC, and the focus of the effort herein, consists of five genes based on a putative precursor peptide with a repeating TVGG motif (Fig. 4). For simplicity, members of the biosynthetic cluster were annotated as *tvgABCDE* (Fig. 4), which, to the best of our knowledge, does not overlap with other gene names. The genes within the pathway encode for the precursor peptide (*tvgA*), a rSAM enzyme (*tvgB*), a putative dioxygenase (*tvgC*), and two putative membrane-associated transporter proteins (*tvgD* and *tvgE*).

### Isolation and characterization of TvgB

**Figure 5:**
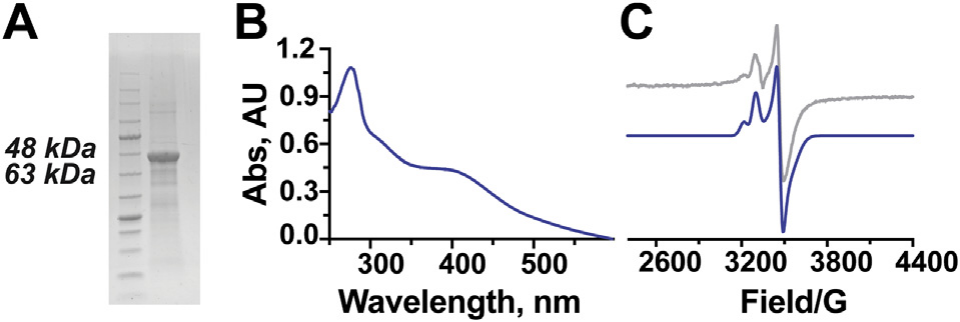
**Characterization of TvgB protein.***A*, an SDS-PAGE showing purified TvgB is nearly homogenous. *B*, the appearance of a 410 nm shoulder in absorbance spectra of reconstituted TvgB suggests the presence of [4Fe-4S] clusters. *C*, X-band (9.3683 GHz) CW spectrum (*gray*) of reduced TvgB at 20 K. Simulation (*blue*) of the CW spectrum using the *g* values of 1.88 (0.0302), 1.93 (0.0057), and 2.03 (0.0135) for the first species and *g* values of 1.90 (0.0352), 1.91 (0.0087), and 2.08 (0.0156) for the second species. (Values in parentheses are the *g* strains). CW, continuous wave.

To validate the bioinformatic findings for Cluster 40, we reconstituted TvgB enzymatic activity with TvgA and structurally characterized the reaction product. The gene corresponding to TvgB was codon optimized for expression in *Escherichia coli*. Recombinant TvgB was coexpressed with pPH151, a plasmid that contains the *suf* operon, to help with iron-sulfur cluster incorporation in TvgB. TvgB was purified to homogeneity (Fig. 5*A*) and chemically reconstituted with iron and sulfur. The appearance of the 410 nm maxima in the UV-Vis absorbance spectrum of reconstituted TvgB (Fig. 5*B*) suggests that iron-sulfur clusters were successfully incorporated. TvgB belongs to a family of rSAM proteins known to bind up to three [4Fe-4S] clusters (34, 35, 36). To determine the potential number of [4Fe-4S] clusters bound by reconstituted TvgB, iron and sulfide quantification was carried out. The analysis revealed that 9.2 ± 0.2 eq of iron and 7.3 ± 0.1 eq of sulfide were present in a single TvgB monomer. Based on this stoichiometry, our current hypothesis is that TvgB contains at least two [4Fe-4S] clusters. In addition, electron paramagnetic resonance (EPR) spectra of TvgB were measured to verify the presence of [4Fe-4S] clusters (Fig. 5*C*). The reduced sample of TvgB was EPR active, resulting in the appearance of two species. The major peak, with *g* values of 1.88, 1.93, and 2.03, is consistent with [4Fe-4S] clusters (37). The signal from a second species was detected in the spectra as well, with *g* values of 1.90, 1.91, and 2.08. The second species could be that of partially degraded Fe-S clusters. Taken together, the UV-Vis absorbance spectra, the iron and sulfur quantification, and the EPR spectra support that TvgB binds at least two [4Fe-4S] clusters.

### Reconstitution of TvgB enzymatic activity

**Figure 6:**
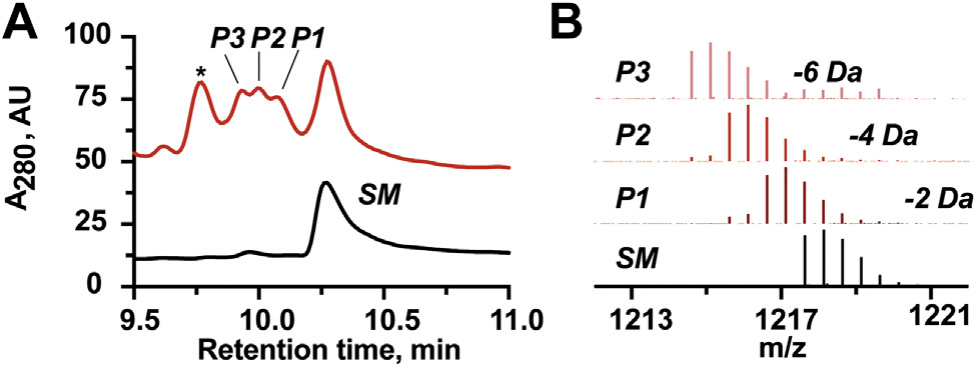
**LCMS data of overnight TvgB reaction with TvgA-4R.***A*, chromatogram of unmodified (*black*) and modified (*red*) peptide TvgA-4R. *B*, HR-MS spectra of [M+2H]^2+^ ions of substrate TvgA-4R and three formed products. ∗denotes an unrelated peak. From the *bottom* to the *top*: TvgA-4R (predicted *m/z* 1218.1337, observed *m/z* 1218.1376), one modification product (2 Da loss, predicted *m/z* 1217.1259, observed *m/z* 1217.1238), two modifications product (4 Da loss, predicted *m/z* 1216.1180, observed *m/z* 1216.1166), and three modifications product (6 Da loss, predicted *m/z* 1215.1102, observed *m/z* 1215.1069).

To demonstrate that TvgB modifies the peptide TvgA, *in vitro* assays were carried out. Initially, constructs of TvgA with various repeats (*e.g.*, two, three, four, etc.) of the TVGG motif were synthesized using microwave-assisted solid state synthesis to identify the minimal active unit. All peptides contained an N-terminal Trp to provide a spectroscopic handle at 280 nm and C-terminal amidation. Purified peptides were reacted with TvgB and an LCMS analysis of the reaction mixture was carried out. As a result of the initial screen, the peptide TvgA-4R containing four repeats of the TVGG motif was chosen for all subsequent analyses. Overnight reactions with equimolar TvgB and TvgA-4R were carried out anaerobically, the reactions were quenched by the addition of 1% TFA, and the soluble fraction was analyzed by RP-HPLC monitoring the absorbance at 280 nm. As shown in Figure 6*A*, following an overnight reaction with TvgB, three additional peaks appear at retention times 9.8 min to 10.1 min. This suggests that TvgB is active with TvgA-4R and could modify the peptide up to three times. To provide support for this hypothesis, the reaction was further analyzed by LC-QToF-HRMS. Mass spectrometry (MS) analysis revealed the presence of [M+2H^+^]^2+^ ions with masses corresponding to the loss of 2 Da (observed *m/z* 1217.1238 amu, 1.7 Δppm), 4 Da (*m/z* 1216.1166 amu, 1.1 Δppm), and 6 Da (*m/z* 1215.1069 amu, 2.7 Δppm) (Fig. 6*B*, *red*, Fig. S3, Table S1) as compared to the unreacted TvgA-4R (*m/z* 1218.1376 amu, 3.2 Δppm, Fig. 6*B*, *black*, Fig. S3, Table S1). These results are consistent with the formation of one, two, and three bonds within the peptide. It should be noted that TvgB can modify synthetic TvgA with up to six TVGG repeats however, due to the limited solubility of the longer peptides, we observed diminishing activity.

### Structural characterization of the TvgB product

**Figure 7:**
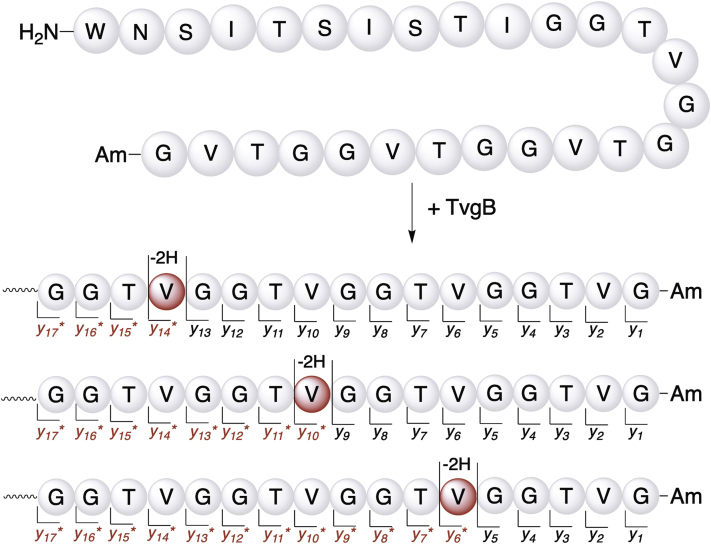
**Localization of the mass loss on the TvgB product.** HR-MS/MS analysis of the TvgB product shows that fragments *y*_*14*_*∗*, *y*_*10*_*∗*, and *y*_*6*_*∗* are 2 Da lighter than the same fragments derived from the substrate. The apparent mixture of modifications to individual valine residues within the TVG repeat suggests the lack of processivity of TvgB under our reaction conditions.

To identify which residue(s) on TvgA that TvgB altered, we used the [M+2H^+^]^2+^ ions of singly modified TvgA in LC-MS/MS analyses since *b*- and *y*-fragments can be used to map where peptide modifications occur. To obtain pure TvgA product with a single modification, TvgB reactions were quenched after 3 h (Fig. S4) and the product peak corresponding to one modification was purified by HPLC. The purified product was subsequently analyzed by LC-MS/MS (Figs. S4 and S5). Our MS/MS analysis of the unmodified and modified TvgA yielded both *b*- and *y*-fragments with near complete amino acid coverage of the peptide (Fig. S6, Tables S2 and S3). Upon analysis of the TvgA product, we found that N-terminal *b*-fragments were left unchanged following the reaction with TvgB. This implied that the loss of 2 Da occurred within the C-terminal repetitive TVGG region as predicted. However, both *b*- and *y*-fragments consistent with no modification and with the loss of 2 Da could be found for repeat-containing fragments. For instance, as shown in Figure 7, if TvgB modified the N-terminal TVGG repeat, we found unmodified *y*-fragments leading up to the Val residue and subsequent *y*-fragments less 2 Da (*y∗*) following Val. This trend was the same for the second and third TVGG motifs (Fig. 7) as well as for *b*-fragments (Fig. S6). While this suggests that only Val residues are being modified, it also suggests that TvgB acts on TvgA in a nonprocessive manner under our reaction conditions. To provide additional MS/MS evidence that Val was being modified, we investigated internal fragments of the modified peptide. We observed that only fragments containing Val have the loss of 2 Da, eliminating the involvement of adjacent Thr and Gly residues in modification (Table S4). Lastly, we synthesized 3G-TvgA-4R where three out of four Val residues were replaced with Gly. As expected, when incubated with TvgB, we observed a maximum of a 2 Da loss in the modified peptide (Table S5). Taken together with the MS/MS analysis, this suggests that Val side chains are site of the hydrogen abstraction by a 5′-deoxyadenosyl radical and possibly the sole site of modification.

**Figure 8:**
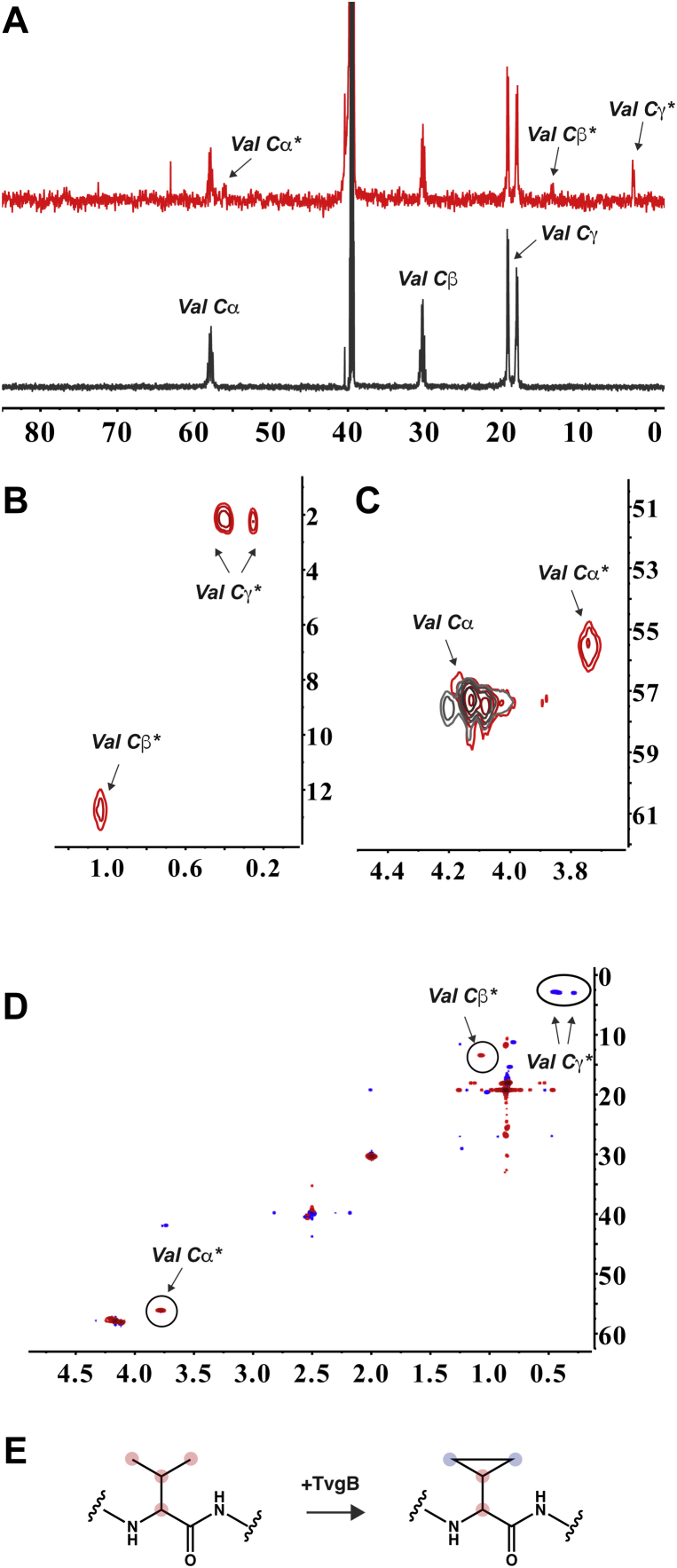
**Structural elucidation of the modified TvgA-4R.***A*, stacked ^13^C NMR spectra for the peptide variant (^13^C_5_^15^N Val)_4_ TvgA-4R (*black*) and TvgB product (*red*). *B* and *C*, ^13^C HSQC strips of overlaid spectra of peptide (*black*) and product (*red*) with definitive peaks revealing formation of C-C bond between Valine methyl groups. *D*, CT-HSQC spectra of modified ^13^C-TvgA-4R product. Positive sign signals are colored in *red* and are indicative of carbons directly bonded to the odd number of C nuclei, carbons that have even number of neighbors have negative sign and appear in *blue*. *E*, a schematic representation of proposed modification catalyzed by TvgB. Carbons that give a rise to signals used in structural elucidation are highlighted and designated. HSQC, heteronuclear single quantum coherence.

To solve the structure of the modification, we carried out the TvgB reaction on a large scale, purified and pooled the reaction products, and conducted 1D/2D NMR analyses. Assessment of the unmodified and modified peptides by ^1^H-^13^C heteronuclear single quantum coherence (^13^C HSQC) NMR and ^1^H-^1^H COSY revealed several new coupled features, including low intensity features in the lower frequency region (<3 ppm *δ*_C_ and <1 ppm *δ*_H_ Figs. S7 and S8). Based on the HR-MS/MS results, we hypothesized that these signals could arise from the nuclei on Val residues that undergo modification. Due to the complexity and poor sensitivity of the natural abundance ^13^C HSQC and COSY NMR spectrum of the TvgB product, we carried out structural elucidation experiments using the isotopically labeled peptide (^13^C_5_^15^N-Val)_4_-TvgA-4R, referred to as ^13^C-TvgA-4R herein. When we compared the ^13^C NMR spectra of the isotopically labeled TvgB product to that of unmodified ^13^C-TvgA-4R, we observed three, low intensity, chemical shifts for carbons on Val (Figs. 8*A* and S9–S13). Since not all four repeats of TvgA-4R are susceptible to TvgB modifications under our reaction conditions, the spectrum of the product contains peaks for unmodified Val as well. In our hands, TvgB modified ^13^C-TvgA-4R an average of 1.2 times per polypeptide, hence there is an ∼3:1 ratio of unmodified ^13^C-Val to modified ^13^C-Val. We assigned the new peak at *δ*_C_ 55.57 ppm to the Cα of ^13^C-Val, a shift from *δ*_C_ 57.29 ppm observed in the substrate. Similarly, we observed a chemical shift of the ^13^C-Val Cβ from *δ*_C_ 29.77 ppm in the substrate to *δ*_C_ 12.80 ppm in the product. Lastly, we observed a new feature at *δ*_C_ 2.26 ppm, which we assigned to the Cγ carbons of ^13^C-Val, a shift from *δ*_C_ 17.44/18.62 ppm in the substrate.

To gain further insight of the structure of modified TvgA-4R, we next performed ^13^C HSQC on ^13^C-TvgA-4R. Differences between the starting material and product spectra were prominent in the upfield region, entirely consistent with the aforementioned peaks observed in the 1D ^13^C NMR spectra. New coupled features at (*δ*_H_ 0.25 ppm; *δ*_C_ 2.26 ppm) and (*δ*_H_ 0.39 ppm; *δ*_C_ 2.26 ppm) were observed in the ^13^C HSQC NMR spectra of the product (Fig. 8*B*). These features were assigned to saturated methylene groups that are bonded (-CH_2_-CH_2_-). Additionally, new features in the ^13^C HSQC spectra of the product appeared at (*δ*_H_ 1.04 ppm; *δ*_C_ 12.80 ppm) and (*δ*_H_ 3.74 ppm; *δ*_C_ 55.57 ppm). These were assigned to Cβ and Cα, respectively (Fig. 8, *B* and *C*). Long-range coupling was also observed in the ^13^C HSQC spectra for these three features (Figs. S9 and S10), suggesting their identification as Cα, Cβ, and Cγ.

**Figure 9:**
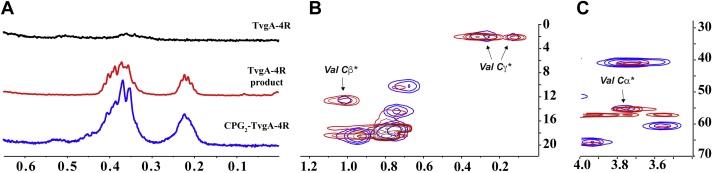
**A comparison of the CPG**_**2**_**-TvgA-4R synthetic peptide to the TvgB product.***A*, stacked ^1^H NMR spectra for unmodified TvgA-4R (*black*, 600 MHz), the TvgB product (*red*, 600 MHz), and synthetic CPG_2_-TvgA-4R (*blue*, 500 MHz). Upfield (*B*) and downfield (*C*) ^13^C HSQC strips of overlaid spectra of the TvgB product (*red*, 600 MHz) and the synthetic peptide CPG_2_-TvgA-4R (*blue*, 500 MHz) with definitive overlapping peaks showing that TvgB catalyzes the formation of CPG from valine. CPG, cyclopropylglycine; HSQC, heteronuclear single quantum coherence.

From the ^13^C HSQC data, we suspected that TvgB catalyzed the formation of cyclopropylglycine (CPG) from Val. Additional support for this hypothesis comes from time-constant HSQC of ^13^C-TvgA-4R product (Fig. 8*D*), where the sign of the signal depends on the parity of directly bonded C nuclei. Indeed, for the modification proposed, all coupled signals match the correct sign: Cα and Cβ have odd number of neighbors thus appearing in red (bonding to the carbonyl carbon is excluded due to C-O decoupling) and Cγ carbons are directly bonded to two other nuclei, appearing with opposite sign in blue (Fig. 8, *E* and *F*). Additionally, the ^1^H and ^13^C chemical shifts observed in both HSQC and ^13^C NMR spectra for the TvgB product are consistent with DFT/parametric hybrid calculated values for the model compound 1-cyclopropyl-1-(methylamino)propan-2-one (Table S6) (38). To validate our hypothesis, we synthesized CPG_2_-TvgA-4R (the mixture of two CPG:2 Val residues were used to closely mimic our product) and characterized it by NMR. The ^1^H NMR spectra of the TvgA-4R substrate, the TvgA-4R product, and the CPG_2_-TvgA-4R control clearly show that both the TvgA-4R product and the CPG_2_-TvgA-4R contained multiplets at *δ*_H_ 0.23 to 0.28 ppm and *δ*_H_ 0.36 to 0.46 ppm (Fig. 9*A*). These features are missing from the unmodified TvgA-4R. The ^13^C HSQC spectra of CPG_2_-TvgA-4R also contain nearly identical coupled features corresponding to the cyclopropyl group (*δ*_H_ 0.24 ppm, *δ*_C_ 2.26 ppm; *δ*_H_ 0.40 ppm, *δ*_C_ 2.26 ppm; and *δ*_H_ 1.04 ppm, *δ*_C_ 12.80; Fig. 9*B*) as well as the coupled feature we assigned to Cα (*δ*_H_ 3.74 ppm; *δ*_C_ 55.57 ppm; Fig. 9*C*).

Taken together, the MS analysis showing the loss of 2, 4, and 6 Da, the localization of the modification within Val residues by MS/MS, 1D and 2D NMR experiments, and the comparison to authentic CPG-containing TvgA indisputably indicates that TvgB installs a carbon-carbon bond in each of the TVGG motifs between the geminal methyl groups on Val side chains.

## Discussion

rSAM-dependent RiPP natural products are proving to have diverse structural topologies derived from the installation of intramolecular bonds catalyzed by the pathway-associated rSAM enzyme. Presently, these topologies are based on the formation of intramolecular cyclophanes, thioether bonds, and ether bonds. Considering that peptides are comprised of 20 natural amino acids, there is an immense possibility that rSAM-dependent RiPP topologies that the community has discovered thus far are the proverbial “tip of the iceberg.” Currently, most rSAM-dependent RiPPs that have been characterized to-date are derived from Haft and Basu’s initial bioinformatic study (10). While many pathways remain uncharacterized from their study, the advancement of bioinformatic toolsets, such as EFI-EST and EFI-GNT, and the recently launched radicalSAM.org (39) has provided opportunities to expand the search for rSAM-dependent RiPPs. For instance, the EST/GNT approach has been used extensively by the Seyedsayamdost group to expand the streptide family of quorum-sensing molecules into new rotapeptides, triglycines, and streptosactins to name a few (33, 40, 41). Therefore, we sought to expand the number of RiPP biosynthetic pathways that require rSAM enzymes.

We focused our efforts on the IPR023867 subfamily of rSAM enzymes since members have previously been annotated as peptide-modifying enzymes. From our SSN/GNT analysis, we noted 22 unexplored and unannotated RiPP biosynthetic pathways. Interestingly, as we analyzed the clusters, two common themes continued to present themselves. First, we found that putative cyclophane-containing peptides are common. For instance, in addition to BGCs associated with Cluster 27 and Cluster 41, we observed similar cyclophane motifs in peptide sequences from BGCs associated with Clusters 19 and 63 (*e.g.*, Aro-Asn and Aro-Xaa-Asn). The second theme is that rSAM enzymes can be found in many nonhomologous RiPP BGCs. For instance, as discussed for Cluster 3, we observed many nonhomologous putative BGCs without precursor peptide sequence overlap. This observation is not isolated to Cluster 3 since nonhomologous BGCs are found to be associated with nearly all SSN clusters (*e.g.*, Cluster 11). While it is tempting to say that each putative RiPP BGC is unique, it is more likely that the association of nonhomologous BGCs to individual clusters is an artifact of the restraints set on the SSN and GNT analysis. In terms of limitations of the SSN analysis, we chose a 40% sequence identity as the restraint to generate nodes. Consequently, nodes contain rSAM enzyme sequences that can be quite divergent, and the representative sequence may not be an accurate description for the entire node. This limitation is propagated in the GNT analysis where the representative sequence was used to define the node’s gene context. Therefore, we assumed that the representative sequence has identical gene context to the remaining nodes which is not the case for every node under our restraints.

Despite these limitations, our assessment of the IPR023867 family aided us in discovering a new RiPP topology based on the formation of CPG through the installation of carbon-carbon bond on unactivated methyl groups. The BGC-associated with Cluster 40 encodes a peptide with a highly conserved TVGG repeating motif. We found this motif to be peculiar since its repeating nature has never been reported before. Therefore, we cloned, expressed, and purified the rSAM associated with the TVG biosynthetic cluster. We reconstituted TvgB enzymatic activity with a truncated TvgA peptide and solved the structure of the modified peptide. By doing so, we discovered a new RiPP topology that is based on the formation of CPG. Interestingly, the installation of CPG by TvgB is repeated multiple times in the precursor peptide. Considering the composition of the biosynthetic cluster, it is likely that the TvgA encodes for multiple small molecule products based on the TVGG motif. We expect that TvgC oxidizes the peptide and that TvgD/TvgE hydrolyze the product and export it to the extracellular matrix. What we described here is, so far, unique to the TVG biosynthetic pathway.

**Figure 10:**
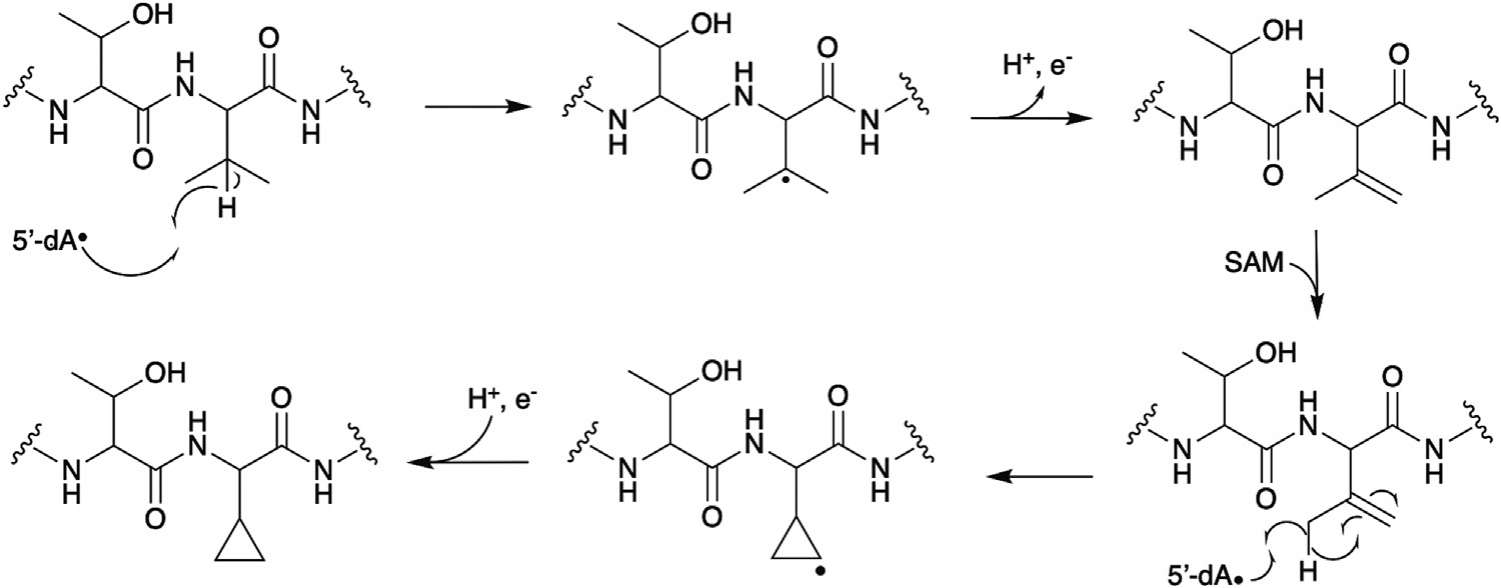
**A schematic representation of the proposed mechanisms for TvgB**.

To install the cyclopropane moiety on unactivated methyl groups of Val, we envision a two-step mechanism (Fig. 10). Logically, at least one Val-C_γ_ must be activated during the reaction course. Therefore, we propose that the 5′-deoxyadenosyl radical derived from a first round of SAM cleavage abstracts a hydrogen from Val-C_β_. The loss of a proton and electron from a Val-C_γ_ results in Val(β,γ-dehydro). In another round of SAM cleavage, we expect the resulting 5′-deoxyadenosyl radical to abstract a hydrogen from the second Val-C_γ_. The resulting resonance stabilized allylic radical, then combines with an electron donated from the Val C_β_-C_γ_ unsaturated bond. The radical would then be quenched by the addition of an electron and a hydrogen, resulting in CPG. This mechanism is reminiscent of the rSAM enzyme MftC. In the biosynthesis of mycofactocin, MftC first catalyzes the oxidative decarboxylation of a C-terminal Tyr yielding an unsaturated tyramine. This is followed by the MftC-catalyzed carbon-carbon bond formation between the C_β_ of the adjacent Val to the C_α_ of the unsaturated tyramine (24). We plan to determine if this mechanism holds true in future studies.

The cyclopropane motif is common in natural products, and examples of their installation by rSAM enzymes is present in literature. For instance, rSAM enzymes are required for the formation of cyclopropanes in the biosynthesis of the spirocyclopropylcyclohexadienone family of antitumor natural products which include yatakemycin and CC-1065 (42, 43, 44). The notable difference in these systems is that they use two components—a HemN-like rSAM enzyme and an SAM-dependent methyltransferase—to achieve the addition of a methylene as well as the formation of the cyclopropane moiety. Our search of the literature revealed that cyclopropane additions by rSAM enzymes are usually coupled to methyl transfer, similar to what was shown for jawsamycin (45). Considering this, it is possible that TvgB is the only known rSAM enzyme that catalyzes the formation of a cyclopropane motif without being coupled to methyl addition. Certainly, TvgB is the first rSAM enzyme to catalyze the formation of a CPG from Val in RiPP maturation.

In summary, we reported on an updated bioinformatic characterization of the rSAM subfamily IPR023867. We describe multiple rSAM-dependent RiPP biosynthetic pathways that, despite being based on known chemical linkages, are expected to result in unprecedented RiPP topologies. Moreover, we made available the resources (*e.g.*, SSN file, GNT files, tables) to broaden the focus of rSAM-dependent RiPP discovery. While these files can be used to outright discover new RiPP topologies, we recommend using them as a springboard to delve deeper into the rSAM cluster families. Lastly, we utilized our SSN results to discover an unprecedented poly-CPG containing RiPP product installed by the rSAM enzyme TvgB. Future work will entail solving the mechanism of TvgB and the product of the pathway. In the decade, since Haft and Basu first described the association of rSAM enzymes to RiPP biosynthesis, nearly two dozen new RiPP topologies have been discovered. With the growing interest in rSAM-dependent RiPPs, we expect that number to explode in the upcoming decade.

## Experimental procedures

### Bioinformatic analysis of IPR023807

A SSN was constructed for the Peptide Modification Radical SAM Enzyme family using EFI-EST (29). The Interpro identifier IPR023807 was used in the Option B method and restricted to the Uniref90 seed sequences. The network was constrained using an E-value of 10^−80^, sequence lengths between 340 and 450 amino acids, and sequence identities of 40%. The resulting network, containing 4131 nodes, was colored automatically by EFI-EST. The network was visualized in Cytoscape (46) and clusters were assigned arbitrary numbers for data handling. Representative sequences from a single cluster were uploaded to EFI-GNT (31) to visualize the gene context. The gene context (±10 genes) for representative sequences from each cluster was analyzed to find a biosynthetic cluster and a precursor peptide sequence. Annotated putative precursor peptide sequences were extracted and aligned using the Uniprot Alignment tool. In cases where the gene context did not have an annotated peptide, we used the NCBI OrfFinder tool to search for a peptide sequence within a 1000 bp window 5′ and 3′ to the associated gene cluster. The potential peptide sequences were also used in the alignment with annotated peptides.

### Preparation of TvgB

The codon optimized L861_16570 gene sequence (Uniprot: S2KHM3) from *Halomonas anticariensis* str. DSM 16096 was synthesized and cloned into the pET-28a vector (Genscript), using the NdeI and XhoI restriction sites. The sequence-verified *tvgB/pET28a* plasmid was cotransformed into *E. coli* BL21 (DE3) competent cells with the plasmid pPH151, which contains the *suf* operon. A single colony of doubly transformed *E. coli* was used to inoculate an overnight culture which, in turn, was used to inoculate 8 l of terrific broth. The cultures were grown at 37 °C with shaking at 200 rpm until an A_600 nm_ of ∼0.8 was achieved. To produce TvgB, 1 mM IPTG, 0.75 g/l of sodium fumarate, and 1× autoinduction metals were added to the media. The temperature was reduced to 21 °C and the cultures were grown overnight. The cells were harvested by centrifugation at 5000*g* for 10 min. All subsequent purification and reconstitution were performed anaerobically (Coy Laboratories). In short, the collected cell pellet was suspended in anaerobic lysis buffer (50 mM Hepes, 300 mM NaCl, 40 mM Imidazole, pH 7.5), supplemented with 1% w/v CHAPS, lysozyme (0.1 mg/g cell paste), and DNase (0.01 mg/g cell paste). The lysate was then transferred to centrifuge tubes and gasket sealed. The lysate was clarified by centrifugation at 20,000*g* for 10 min at 4 °C. The lysate was transferred back into the chamber and subsequently loaded onto a 5 ml HisTrap HP column. The column was washed with 25 ml lysis buffer and TvgB was eluted with elution buffer (50 mM Hepes, 300 mM NaCl, 300 mM Imidazole, pH 7.5). Purified TvgB was buffer exchanged into storage buffer (50 mM Hepes, 300 mM NaCl, 10 mM DTT, 10% glycerol, pH 7.5) using a PD-10 column (GE Healthcare). To reconstitute TvgB, 10 mM DTT and 12 M equivalents of both FeCl_3_ and Na_2_S were added to the protein solution and stirred at room temperature (rt) for 30 min. The reconstituted enzyme buffer exchanged for a PD-10 column, concentrated using 30 kDa spin column, aliquoted, and flash frozen for future use.

### Iron and sulfide quantification

TvgB was precipitated with 3 M trichloroacetic acid and pellet was used to determine the enzyme concentration *via* Bradford assay, while supernatant was subject to iron and sulfur quantification studies. A ferrozine-based assay was used to determine iron content, where the formation of ferrozine–iron complex was monitored at 562 nm with ε = 27.9 mM^−1^ cm^−1^. In short, 100 μl of 20 μM protein was diluted with 330 μl of diH_2_O and mixed with 20 μl of sodium ascorbate, 20 μl of 10 mM ferrozine, and 20 μl of saturated sodium acetate. Sulfur concentration was quantified by its incorporation into methylene blue, in the presence of acidic N, N-dimethyl-phenylenediamine and ferric chloride. For that, 1% (w/v) zinc acetate and 50 μl of 7% (w/v) sodium hydroxide were added to 200 μl of 10 μM TvgB. After 15 min of incubation at room temperature, 150 μl of 0.1% (w/v) N, N-dimethyl-p-phenylenediamine (in 5 M HCl) and 150 μl of 10 mM FeCl_3_ (in 1 M HCl) were added to the mixture. The solution was thoroughly mixed and incubated at room temperature for another 20 min. The methylene blue absorbance was measured at 670 nm (ε = 34.5 mM^−1^ cm^−1^).

### EPR spectroscopy analysis

Reduction of the iron-sulfur clusters was achieved by addition of excess (5 mM) dithionite to 600 μM TvgB in storage buffer. Samples were transferred into 4 mm OD quartz tubes in the inert atmosphere chamber and flash frozen in liquid nitrogen. Tubes were temporarily capped with clamped tygon tubing, removed from the chamber, partially evacuated, back-filled with a partial pressure of helium (100 mTorr) to facilitate thermal equilibration, and flame-sealed. Samples were kept frozen in liquid nitrogen for a few hours, until they were inserted into a precooled resonator. Continuous wave EPR spectra at 20 K were acquired at 9.3683 GHz on a Bruker E580 spectrometer with an SHQE resonator and equipped with a Bruker/ColdEdge Stinger cryogenic system. Spectra were acquired with 4 G modulation amplitude at 100 kHz, 2000 G scan width, a time constant of 81 ms, sweep time of 84 s with a 7 s delay to allow field settling at the end of each scan, and signal averaging of four scans. The microwave power was selected to be in a range where the signal increases linearly with square root of power. Simulations were performed using the Bruker BioSpin software Aniso-spin fit. The g values for two overlapping signals were adjusted manually using values reported for MftC (24) as the starting points.

### Synthesis and purification of substrate TvgA-4R and its variants

Fmoc-based solid phase syntheses of C-amidated peptide TvgA-4R and its variants were performed using CEM Liberty Blue Automated Microwave Peptide Synthesizer. Rink Amide ProTide (LL) resin 100 to 200 mesh (0.18 mmol/g) and standard Fmoc and tBu-protected amino acids were used for TvgA-4R synthesis at 0.05 mmol scale. A 10% piperazine (w/v) solution of 10% EtOH, 88% NMP, and 2% DBU was utilized for Fmoc deprotection reactions. The activating reagent, consisted of 0.25 M DIC in DMF, was used for Fmoc amino acids coupling, and 0.5 M Oxyma in DMF was used as an activator base. Single coupling reactions were applied to all amino acids using standard microwave conditions. Once the synthesis was completed, the resin was washed twice with methanol and once with chloroform, then dried under the vacuum for 30 min. Next, the peptide was cleaved from the resin *via* incubation with cleavage cocktail (95% TFA, 2.5% TIS, and 2.5% H_2_O, 30 ml/g) for 30 min at 38 °C. After filtering the mixture to remove cleaved resin, the filtrate was dropwise introduced into the ice-cold 20 ml of diethyl ether. The suspension was left at a room temperature for 1 h allowing white precipitate to form. The solution was centrifuged for 10 min at 5000*g*, 4 °C and diethyl ether layer was discarded. The precipitated peptide was dried from the ether residuals and resuspended in 30% dimethylsulfoxide (DMSO, in 0.1% TFA). Gentle heating in water bath was applied to help dissolve the pellet. Next, the crude peptide was loaded onto semi-preparative 10 × 250 mm C4 15 to 20 μm reverse-phase column (Vydac 214TP152010) using a Shimadzu UFLC with 0.1% TFA and acetonitrile as mobile phases. The peptide fraction was collected, verified by LC-MS, and lyophilized overnight.

### Peptide modification reactions

Reactions were set up in anaerobic conditions with all the reagents prepared in the chamber. Previously lyophilized TvgA-4R was transferred to the chamber and suspended in DMSO. The concentrations of TvgA-4R were determined spectroscopically monitoring absorbance at 280 nm (ε = 5.6 mM^−1^ cm^−1^). TvgAB reactions were set up with sequential addition of 200 μM TvgA-4R, 2 mM SAM, 2 mM sodium dithionite, 2 mM DTT, and 200 μM TvgB in reaction buffer (50 mM Hepes, 200 mM KCl, 10 mM DTT, 10% glycerol, pH 7.5). The reactions were carried out overnight at room temperature and quenched with 1% TFA. After centrifugation, the supernatant was injected on a 4.6 × 150 mm C_8_ 5 μm column (Symmetry) using 0.1% formic acid (Buffer A) and 100% acetonitrile (Buffer B) as the mobile phases. Wavelengths at 254 nm and 274 nm were monitored, and the chromatogram was reported at 274 nm.

### LC-QToF-MS and MS/MS analysis of peptide

All mass spectrometry experiments were carried out on Shimadzu Prominence-i LC-2030 HPLC coupled to a Shimadzu LCMS 9030. Intact mass analysis was performed using the positive ESI mode with voltage of 4 kV, with a scan *m/z* range 200 to 2000 amu. Tandem analysis was carried out on parent ions between 1217.1 amu to 1219.1 amu, using a collision energy of 80 keV, and a mass range of 200 to 2000 amu. The data were analyzed using the Shimadzu Insight Explorer program and the UCSF Protein Prospector web server.

### NMR analyses of TvgA-4R and its product

For the NMR analysis, the starting material and the product lyophilized samples were dissolved in 99.9% deuterated DMSO. NMR spectra were recorded on Bruker Avance Neo 600 MHz spectrometer at the CU Anschutz Medical Campus. Time constant ^13^C HSQC experiments were carried out on a Bruker Avance Neo 900 MHz spectrometer at the CU Anschutz Medical Campus. Additional NMR analyses, specifically those for CPG_2_-TvgA-4R, were carried out on a 500 MHZ Bruker Avance Neo. All spectra were processed using TopSpin v. 2.1 program (Bruker) and analyzed using MestReNova v. 10.0.1 program (Mestrelab Research).

## Data availability

All data are contained within the article.

## Supporting information

This article contains supporting information.

## Conflict of interest

The authors declare that they have no conflicts of interest with the contents of this article.
