# Supplementary material for: Identification of a poly-cyclopropylglycine–containing peptide via bioinformatic mapping of radical S-adenosylmethionine enzymes
Source: J Biol Chem. 2022 Mar 31;298(5):101881. doi: 10.1016/j.jbc.2022.101881 (PMC9062424; doi:10.1016/j.jbc.2022.101881)

**Supporting Information**

Bioinformatic mapping of radical S-adenosylmethionine enzymes leads to the discovery of a poly-cyclopropylglycine containing peptide.

Anastasiia Kostenko^1^, Yi Lien^1^, Aigera Mendauletova^1^, ﻿Thacien Ngendahimana^1^, Ivan Novitskiy^1^, Sandra S. Eaton^1^, and John A. Latham^1*^

^1^Department of Chemistry and Biochemistry, University of Denver, Denver, Colorado 80210, United States

*Corresponding author: [john.latham@du.edu](mailto:john.latham@du.edu)

**Contents**:

Page 2 **Figure S1.** The sequence similarity network of rSAM enzymes family IPR023807 created using Uniref 90 database revealed 118 clusters.

Page 3 **Figure S2.** Biosynthetic gene cluster diagrams and amino acid composition of their assumptive precursor peptides core region.

Page 4 **Figure S3.** HR-LC-MS data of the overnight reaction of TvgB and substrate TvgA-4R.

Page 4 **Table S1**. MS ions of starting material (SM) TvgA-4R and TvgA-4R* products isolated from overnight reaction with TvgB.

Page 5 **Figure S4.** LC-MS analyses of substrate TvgA-4R and TvgB product with single modification collected after 1h reaction.

Page 5 **Figure S5**. HR-LC-MS/MS data of substrate TvgA-4R and TvgB product with one modification.

Page 5 **Figure S6**. HR-LC-MS/MS fragmentation pattern of substrate TvgA-4R and TvgB products with one and two modifications.

Page 6 **Table S2.** The *b-* and *y-* fragment ions of unmodified peptide TvgA-4R detected in HR-MS/MS experiments.

Page 7 **Table S3**. The *b-* and *y-* fragment ions of modified peptide TvgA-4R* detected in HR-MS/MS experiments.

Page 8 **Table S4**. Internal *b* fragment ions that contain single modification (-2Da).

Page 8 **Table S5**. Sequences of substrate variants used in this study.

Page 9 **Figure S7**. ^13^C HSQC NMR spectra of the substrate peptide TvgA-4R and TvgB product.

Page 10 **Figure S8.** COSY NMR spectra of the TvgA-4R before and after the reaction with TvgB.

Page 11 **Figure S9.** ^1^H, ^13^C, ^13^C HSQC NMR spectra of the labelled unmodified peptide ^13^C Val TvgA-4R.

Page 13 **Figure S10.** ^1^H, ^13^C, ^13^C HSQC NMR spectra of the labelled modified peptide ^13^C Val TvgA-4R*.

Page 16 **Figure S11.** Overlayed ^13^C HSQC of TvgA-4R and ^13^C Val TvgA-4R variant before and after the reaction with TvgB.

Page 18 **Table S6**. ^1^H and ^13^C Experimental and DFT calculated chemical shifts for ^13^C Val TvgA-4R.

Page 18 **Figure S12.** ^13^C HSQC NMR spectra of the synthesized CPG_2_ TvgA-4R variant.

Page 19 **Figure S13.** ^13^C HSQC NMR spectra of the modified ^13^C_4_ ^15^N Thr_4_ TvgA-4R variant.

**Figure S1.** The sequence similarity network of rSAM enzymes family IPR023867 created using Uniref 90 database with the annotated 118 clusters.

**
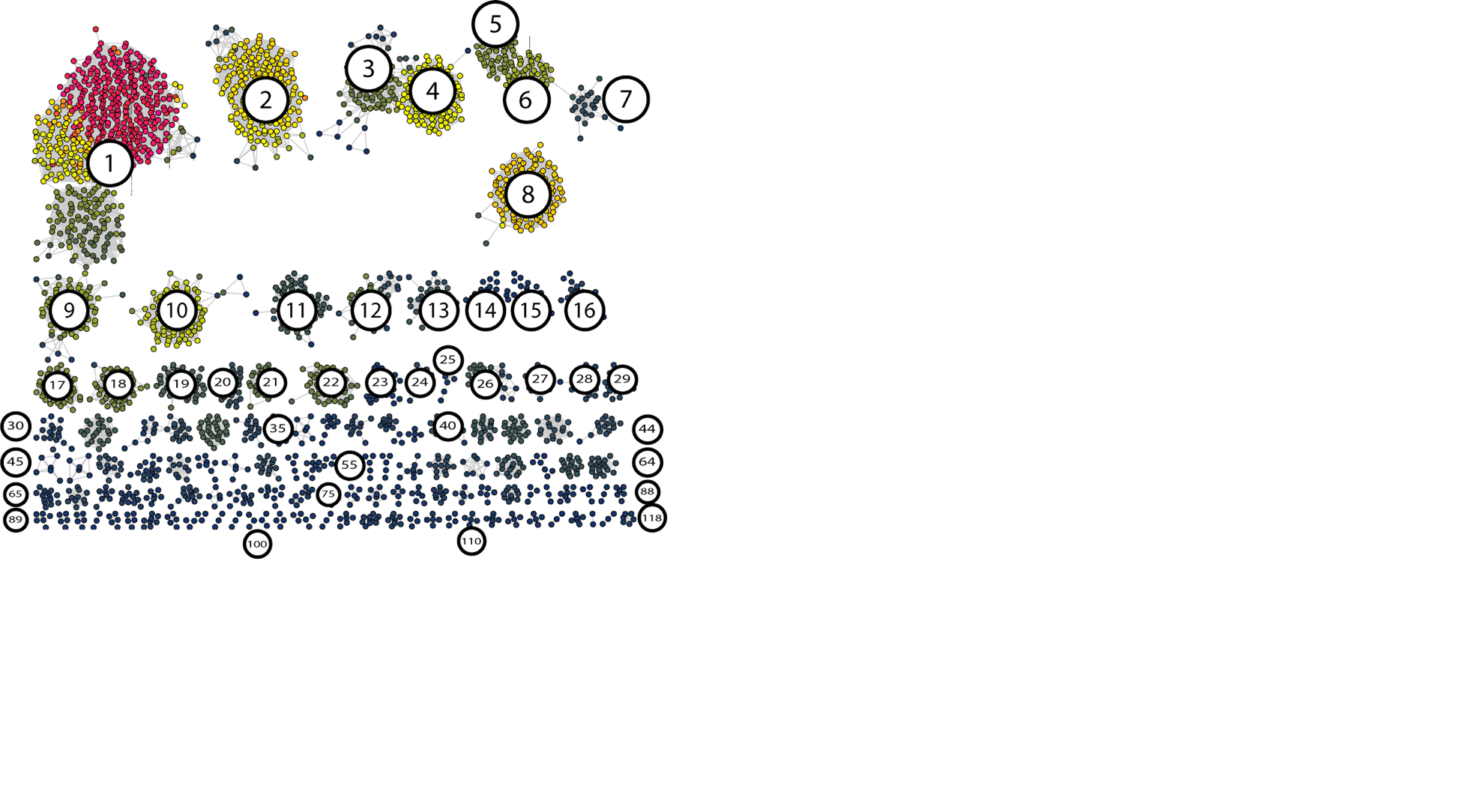
**

**Figure S2.** Biosynthetic gene cluster diagrams and amino acid composition of their assumptive precursor peptides core region. Peptides from distinct pathways were analyzed and chosen as a representative sequences that have unique novel features.

**
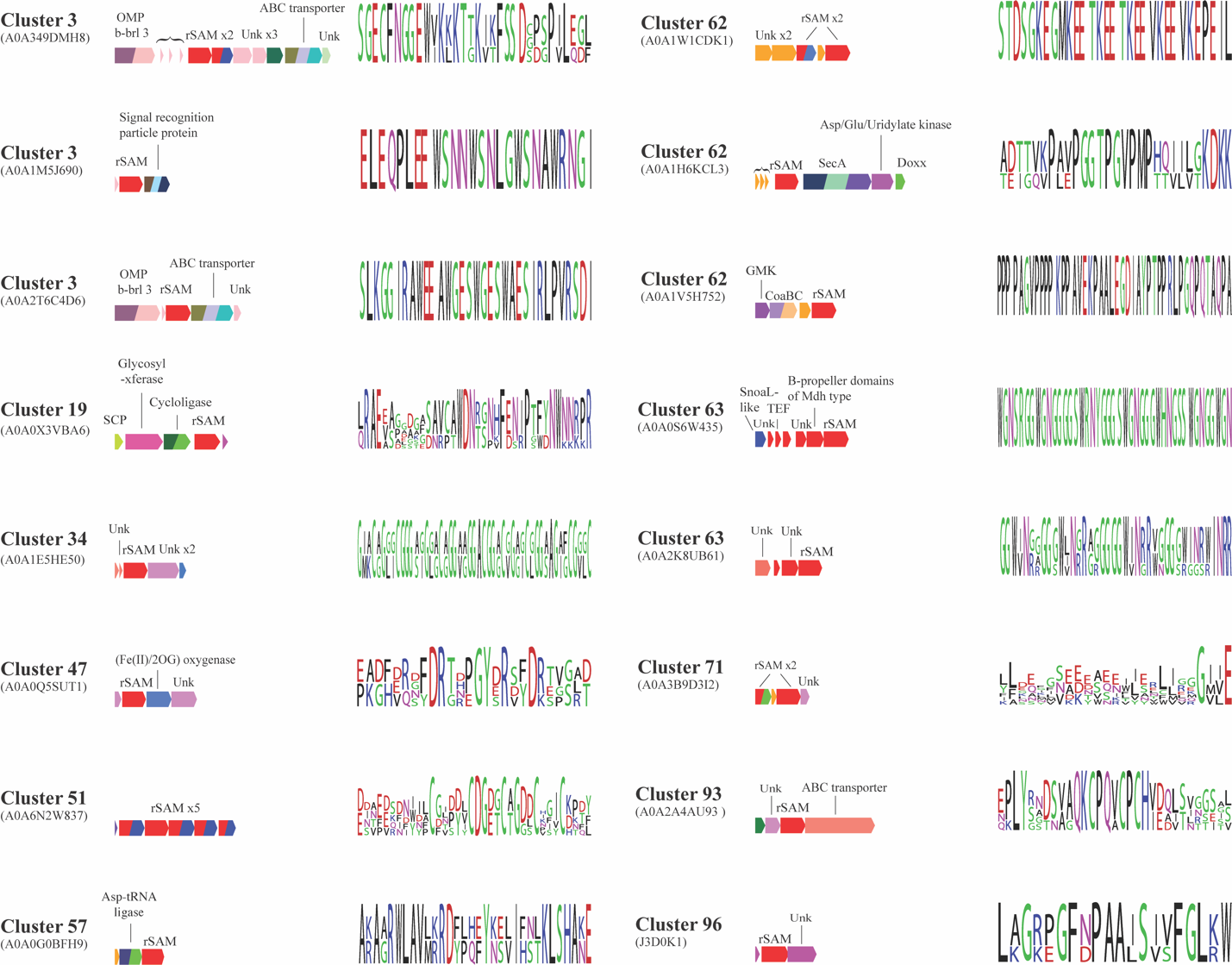
**

**Figure S3.** HR-LC-MS data of the overnight reaction of TvgB and substrate TvgA-4R. **A)** Total ion chromatograms of the overall reaction; *inset*: amplified image of the mixture of starting material and formed products (70% conversion). **B)** Extracted ion chromatograms corresponding to the (M+2H)^2+^ ions of substrate TvgA-4R (*blue*), products with 1, 2, and 3 modifications (*purple*).


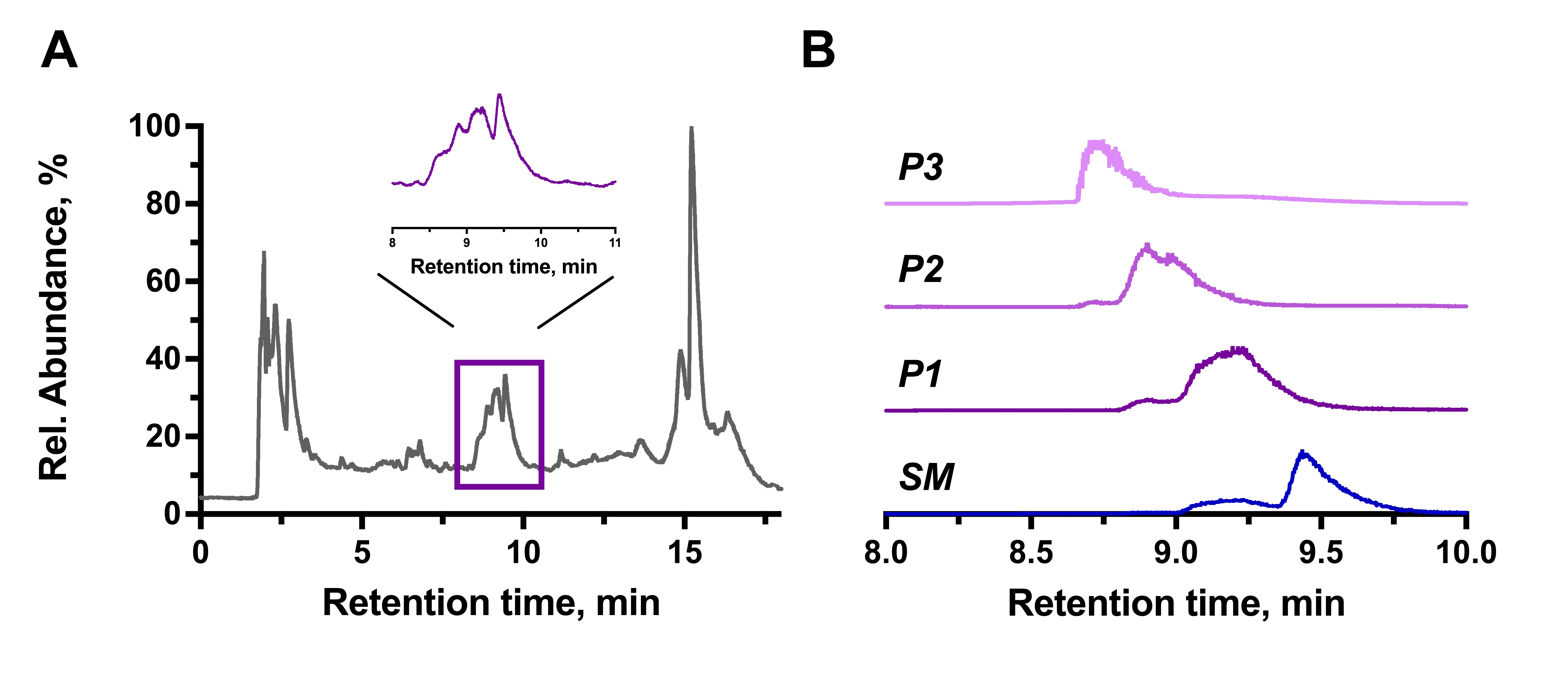


**Table S1 –** MS ions of starting material (SM) TvgA-4R and TvgA-4R* products isolated from overnight reaction with TvgB. Ions with 2 Da, 4 Da, and 6 Da lighter masses compared to unreacted TvgA-4R are consistent with the one, two and three modifications on the peptide.

|  | Charge state ^2+^ | | | Charge state ^3+^ | | |
| --- | --- | --- | --- | --- | --- | --- |
|  | **Observed *m/z*** | **Predicted *m/z*** | **Δ ppm** | **Observed *m/z*** | **Predicted *m/*z** | **Δ ppm** |
| TvgA-4R  SM | 1218.1376 | 1218.1337 | 3.2 | 812.7608 | 812.7608 | 0.1 |
| TvgA-4R*  1 modification | 1217.1238 | 1217.1259 | 1.7 | 811.7527 | 811.7530 | 0.4 |
| TvgA-4R*  2 modifications | 1216.1166 | 1216.1180 | 1.1 | 811.0795 | 811.0811 | 2.0 |
| TvgA-4R*  3 modifications | 1215.1069 | 1215.1102 | 2.7 | 810.4066 | 810.4097 | 3.8 |

**Figure S4.** LC-MS analyses of substrate TvgA-4R and TvgB product with single modification collected after 1h reaction. **A)** Elution time comparison of pure substrate and product: unmodified peptide TvgA-4R elutes later (9.35 minutes, *grey*) than modified peptide (9.11 minutes, *red*). **B)** HR-MS spectrum of [M+3H]^+3^ (*m/z* 812.7608) and [M+2H]^+2^ (*m/z* 1218.1376) ions of substrate TvgA-4R. **C)** HR-MS spectrum of [M+3H]^+3^  (*m/z* 811.7527) and [M+2H]^+2^ (*m/z* 1217.1238) ions of modified TvgA-4R, indicating 2 Da loss.

**Figure S5**. **A)** HR-LC-MS/MS data of substrate TvgA-4R. **B)** HR-LC-MS/MS data of TvgB product with one modification. The *b-* fragments are highlighted in *red*, *y-* fragments are highlighted in *blue*.

**
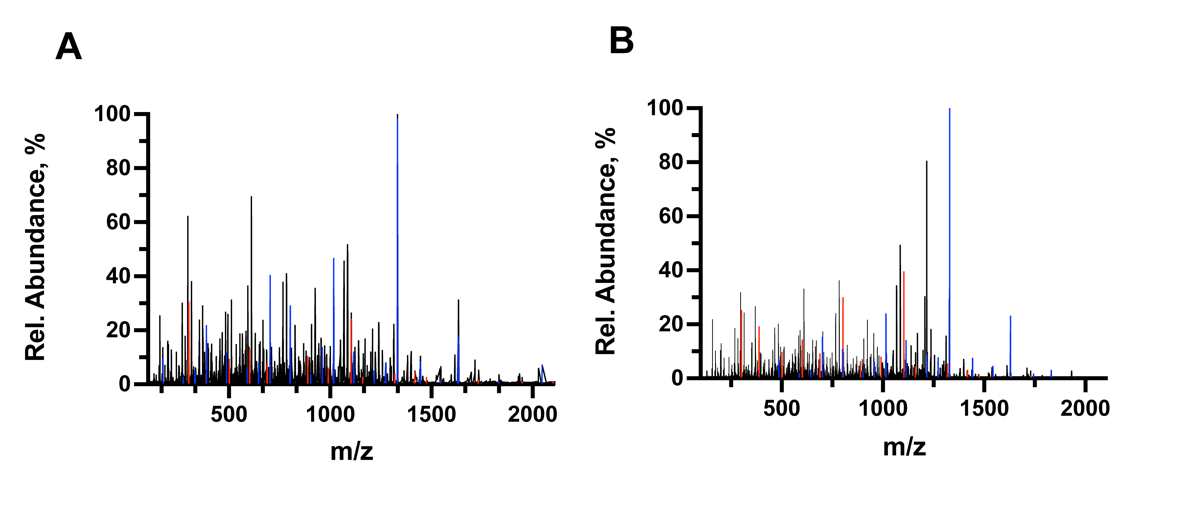
**

**Figure S6**. HR-LC-MS/MS fragmentation pattern of substrate TvgA-4R and TvgB products with one and two modifications. Fragments with one and two asterisks are indicative of the loss of 2 Da and 4 Da respectively.

**
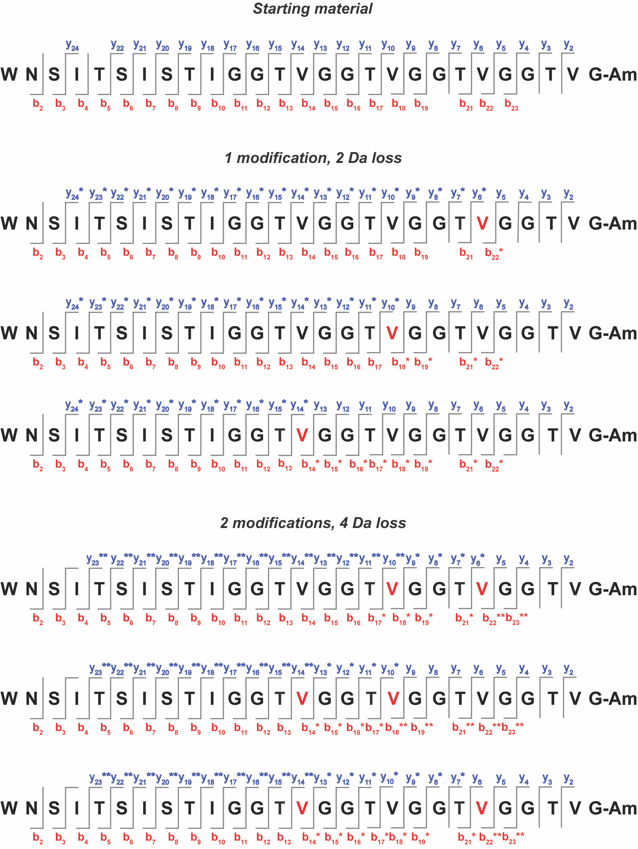
**

**Table S2 –** The *b-* and *y-* fragment ions of unmodified peptide TvgA-4R detected in HR-MS/MS experiments. [M+2H]^2+^ ion with *m/z* 1218.1376 was chosen as precursor ion.

| *y-*ions | Observed  *m/z* | Theoretical  *m/z* | Δ ppm | *b-*ions | Observed  *m/z* | Theoretical  *m/z* | Δ ppm | |
| --- | --- | --- | --- | --- | --- | --- | --- | --- |
| *y_2_* | 174.1245 | 174.1237 | 4.6 | ***b_2_*** | 301.1299 | 301.1295 | 1.3 |  |
| *y_3_* | 275.1699 | 275.1714 | 5.5 | ***b_3_*** | 388.1614 | 388.1615 | 0.3 |  |
| *y_4_* | 332.1925 | 332.1928 | 0.9 | ***b_4_*** | 501.2450 | 501.2456 | 1.2 |  |
| *y_5_* | 389.2144 | 389.2143 | 0.3 | ***b_5_*** | 602.2944 | 602.2933 | 1.8 |  |
| *y_6_* | 488.2826 | 488.2827 | 0.2 | ***b_6_*** | 689.3246 | 689.3253 | 1.0 |  |
| *y_7_* | 589.3251 | 589.3304 | 9.0 | ***b_7_*** | 802.4125 | 802.4094 | 3.9 |  |
| *y_8_* | 646.3523 | 646.3519 | 0.6 | ***b_8_*** | 889.4485 | 889.4414 | 8.0 |  |
| *y_9_* | 703.3744 | 703.3733 | 1.6 | ***b_9_*** | 990.4953 | 990.4891 | 6.3 |  |
| *y_10_* | 802.4456 | 802.4417 | 4.9 | ***b_10_*** | 1103.5765 | 1103.5732 | 3.0 |  |
| *y_11_* | 903.4912 | 903.4894 | 2.0 | ***b_11_*** | 1160.6004 | 1160.5946 | 5.0 |  |
| *y_12_* | 960.5125 | 960.5109 | 1.7 | ***b_12_*** | 1221.6214 | 1217.6161 | 4.3 |  |
| *y_13_* | 1017.5333 | 1017.5323 | 1.0 | ***b_13_*** | 1318.6686 | 1318.6638 | 3.6 |  |
| *y_14_* | 1116.6076 | 1116.6008 | 6.1 | ***b_14_*** | 1417.7336 | 1417.7322 | 1.0 |  |
| *y_15_* | 1217.6354 | 1217.6484 | 10.7 | ***b_15_*** | 1474.7574 | 1474.7536 | 2.6 |  |
| *y_16_* | 1274.6706 | 1274.6699 | 0.6 | ***b_16_*** | 1531.7771 | 1531.7751 | 1.3 |  |
| *y_17_* | 1331.6939 | 1331.6914 | 1.9 | ***b_17_*** | 1632.8458 | 1632.8228 | 14.1 |  |
| *y_18_* | 1444.7749 | 1444.7754 | 0.4 | ***b_18_*** | 1731.9097 | 1731.8912 | 10.7 |  |
| *y_19_* | 1545.8143 | 1545.8231 | 5.7 | ***b_19_*** | 1788.9039 | 1788.9127 | 4.9 |  |
| *y_20_* | 1632.8525 | 1632.8551 | 1.6 | ***b_21_*** | 1946.9784 | 1946.9818 | 1.7 |  |
| *y_21_* | 1745.9325 | 1745.9392 | 3.8 | ***b_23_*** | 2103.0632 | 2103.0717 | 4.1 |  |
| *y_22_* | 1832.9537 | 1832.9712 | 9.6 |  |  |  |  |  |
| *y_24_* | 2047.0535 | 2047.1030 | 24.2 |  |  |  |  |  |
|  |  |  |  |  |  |  |  |  |

**Table S3 –** The *b-* and *y-* fragment ions of modified peptide TvgA-4R* detected in HR-MS/MS experiments. [M+2H]^2+^ ion of TvgA-4R* with single modification (*m/z* 1217.1238) was chosen as precursor ion. *y***-*ions of modified TvgA-4R* are 2 Da lighter than *y-*fragments. Fragmentation data for product with modification in the second TVGG repeat was used for representation.

| *y-*ions | Observed  *m/z* | Theoretical  *m/z* | Δ ppm | *b-*ions | Observed  *m/z* | Theoretical  *m/z* | Δ ppm |
| --- | --- | --- | --- | --- | --- | --- | --- |
| *y_2_* | 174.1233 | 174.1237 | 2.5 |  |  |  |  |
| *y_3_* | 275.1708 | \| 275.1714 \| \| --- \| | 2.2 | ***b_2_*** | 301.1288 | 301.1295 | 2.3 |
| *y_4_* | 332.1923 | 332.1928 | 1.6 | ***b_3_*** | 388.1613 | 388.1615 | 0.5 |
| *y_5_* | 389.2103 | 389.2143 | 10.3 | ***b_4_*** | 501.2458 | 501.2456 | 0.4 |
| *y_6_* | 488.2813 | 488.2827 | 2.8 | ***b_5_*** | 602.2941 | 602.2933 | 1.3 |
| *y_7_* | 589.3249 | 589.3304 | 9.3 | ***b_6_*** | 689.3271 | 689.3253 | 2.6 |
| *y_8_* | 646.3499 | 646.3519 | 3.1 | ***b_7_*** | 802.4105 | 802.4094 | 1.4 |
| *y_9_* | 703.3712 | 703.3733 | 3.0 | ***b_8_*** | 889.4417 | 889.4414 | 0.3 |
| *y_10_^*^* | 800.4219 | 800.4261 | 5.2 | ***b_9_*** | 990.4901 | 990.4891 | 1.0 |
| *y_11_^*^* | 901.4738 | 901.4738 | 4.0 | ***b_10_*** | 1103.5735 | 1103.5732 | 0.3 |
| *y_12_^*^* | 958.4944 | 958.4952 | 0.8 | ***b_11_*** | 1160.5866 | 1160.5946 | 0.5 |
| *y_13_^*^* | 1015.5162 | 1015.5167 | 0.6 | ***b_12_*** | 1217.6075 | 1217.6161 | 7.1 |
| *y_14_^*^* | 1114.5827 | 1114.5851 | 2.1 | ***b_13_*** | 1318.6634 | 1318.6638 | 0.3 |
| *y_15_^*^* | 1215.6225 | 1215.6328 | 8.5 | ***b_14_*** | 1417.7321 | 1417.7322 | 0.1 |
| *y_16_^*^* | 1272.6541 | 1272.6543 | 0.2 | ***b_15_*** | 1474.7537 | 1474.7536 | 0.1 |
| *y_17_^*^* | 1329.6757 | 1329.6757 | 0.1 | ***b_16_*** | 1531.7747 | 1531.7751 | 0.3 |
| *y_18_^*^* | 1442.7590 | 1442.7598 | 0.6 | ***b_17_*** | 1632.8296 | 1632.8228 | 4.2 |
| *y_19_^*^* | 1543.8034 | 1543.8075 | 2.6 | ***b_18_****^*^* | 1729.8679 | 1729.8755 | 4.4 |
| *y_20_^*^* | 1630.8391 | 1630.8395 | 0.3 | ***b_19_****^*^* | 1786.9015 | 1786.8970 | 2.5 |
| *y_21_^*^* | 1743.9239 | 1743.9236 | 0.2 | ***b_21_****^*^* | 1944.9623 | 1944.9661 | 1.9 |
| *y_23_^*^* | 1830.9565 | 1830.9556 | 0.5 | ***b_22_****^*^* | 2044.043 | 2044.0346 | 4.1 |
| *y_24_^*^* | 2045.0943 | 2045.0873 | 3.4 |  |  |  |  |

**Table S4.** Internal *b* fragment ions that contain single modification (-2 Da).

| Sequence | Observed *m/z* | Theoretical *m/z* | Δ ppm |
| --- | --- | --- | --- |
| VG | 155.0808 | 155.0815 | 4.3 |
| TV | 199.1078 | 199.1077 | 0.4 |
| VGG | 212.1022 | 212.1030 | 3.6 |
| GTV/TVG | 256.1278 | 256.1292 | 5.4 |
| GGTV/VGGT/TVGG/GTVG | 313.1489 | 313.1506 | 5.6 |
| GGTVG/GTVGG | 370.1693 | 370.1721 | 7.6 |
| GGTVGG | 427.1950 | 427.1936 | 3.4 |
| GTVGGT | 471.2163 | 471.2198 | 7.3 |

**Table S5 –** Sequences of substrate variants used in this study. All the peptides are amidated at C-terminus.

| **TvgA-4R** | WNSITSISTIGGTVGGTVGGTVGGTVG-Am | up to -6 Da |
| --- | --- | --- |
| **TvgA-3R** | WNSITSISTIGGTVGGTGGTVGGTG-Am | up to -6 Da |
| **TvgA-2R** | WNSITSISTIGGTVGGTVGG-Am | no reaction |
| **TvgA-6R** | WNSITSISTIGGTVGGTVGGTVGGTVGGTVGGTVGGTVGG-Am | reactive  (high hydrophobicity) |
| **TvgA-1_63** | WNSITSISTIGGTVGGTVGGTVGGTVGGTVGGTVGGTVGGTVGGTVGGTVSGTVGGTSAVGGL-Am | reactive  (high hydrophobicity) |
| **^13^C_5_ ^15^N Val_4_ TvgA-4R** | WNSITSISTIGGTVGGTVGGTVGGTVG- Am | up to -6 Da |
| **^13^C_4_ ^15^N Thr_4_ TvgA-4R** | WNSITSISTIGGTVGGTVGGTVGGTVG-Am | up to -6 Da |
| **(CPG)_2_ TvgA-4R** | WNSITSISTIGGT(CPG)GGT(CPG)GGTAGGTAG-Am | - |
| **(V to A)_4_ TvgA-4R** | WNSITSISTIGGTAGGTAGGTAGGTAG-Am | no reaction |
| **(V to G)_3_ TvgA-4R** | - WNSITSISTIGGTVGGTGGGTGGGTGG-Am - WNSITSISTIGGTGGGTVGGTGGGTGG-Am - WNSITSISTIGGTGGGTGGGTVGGTGG-Am - WNSITSISTIGGTGGGTGGGTGGGTVG-Am | -2 Da each |

**Figure S7**. ^13^C HSQC NMR spectra of the **A)** substrate peptide TvgA-4R; **B)** TvgB product in deuterated DMSO recorded on Bruker 500 MHz.

A)


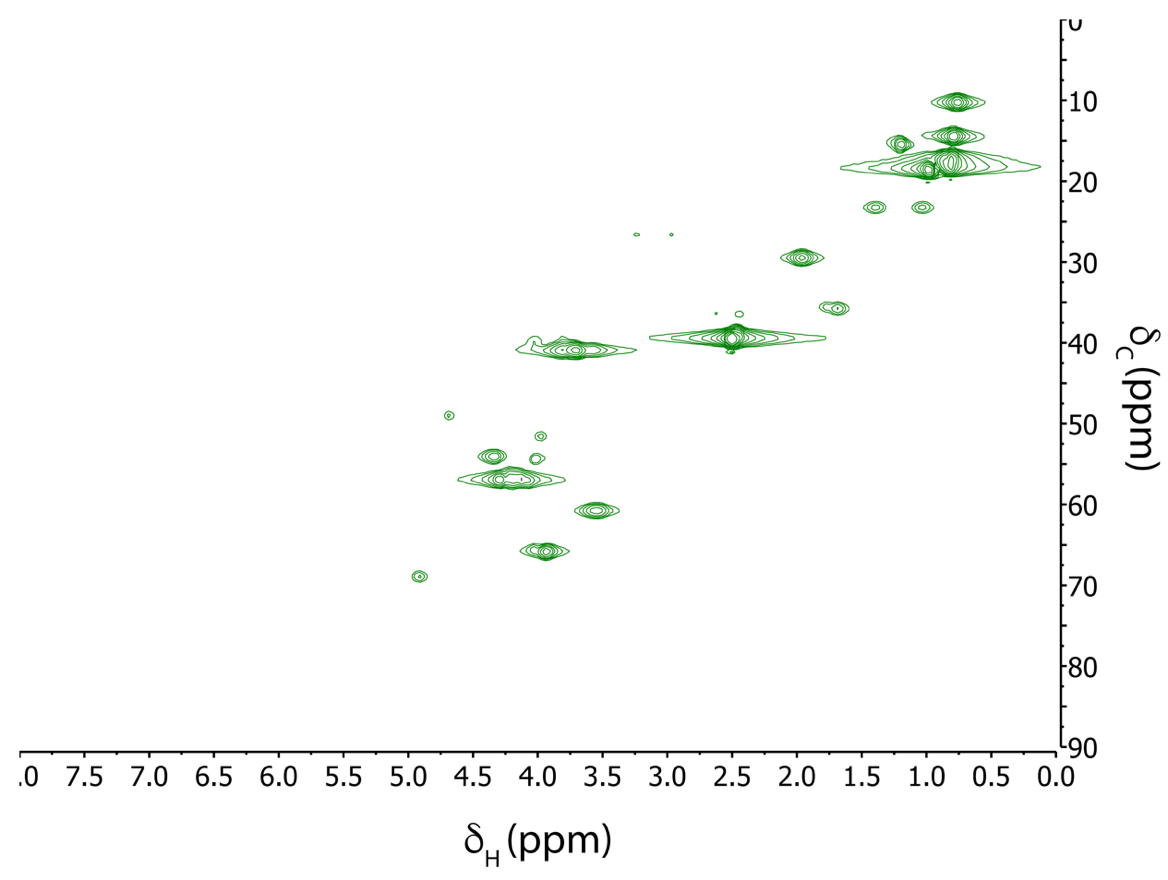


B)


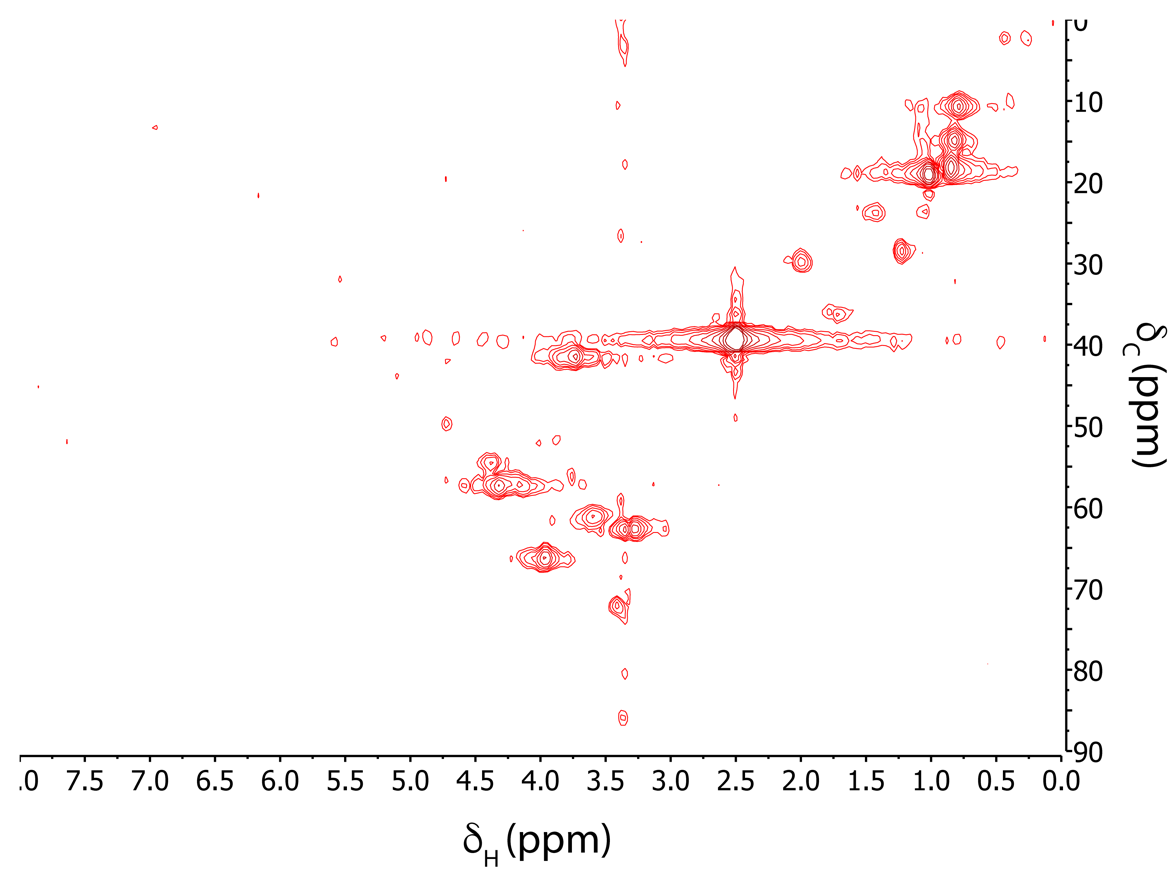


**Figure S8.** A) COSY NMR of starting material; B) COSY NMR of product.

A)


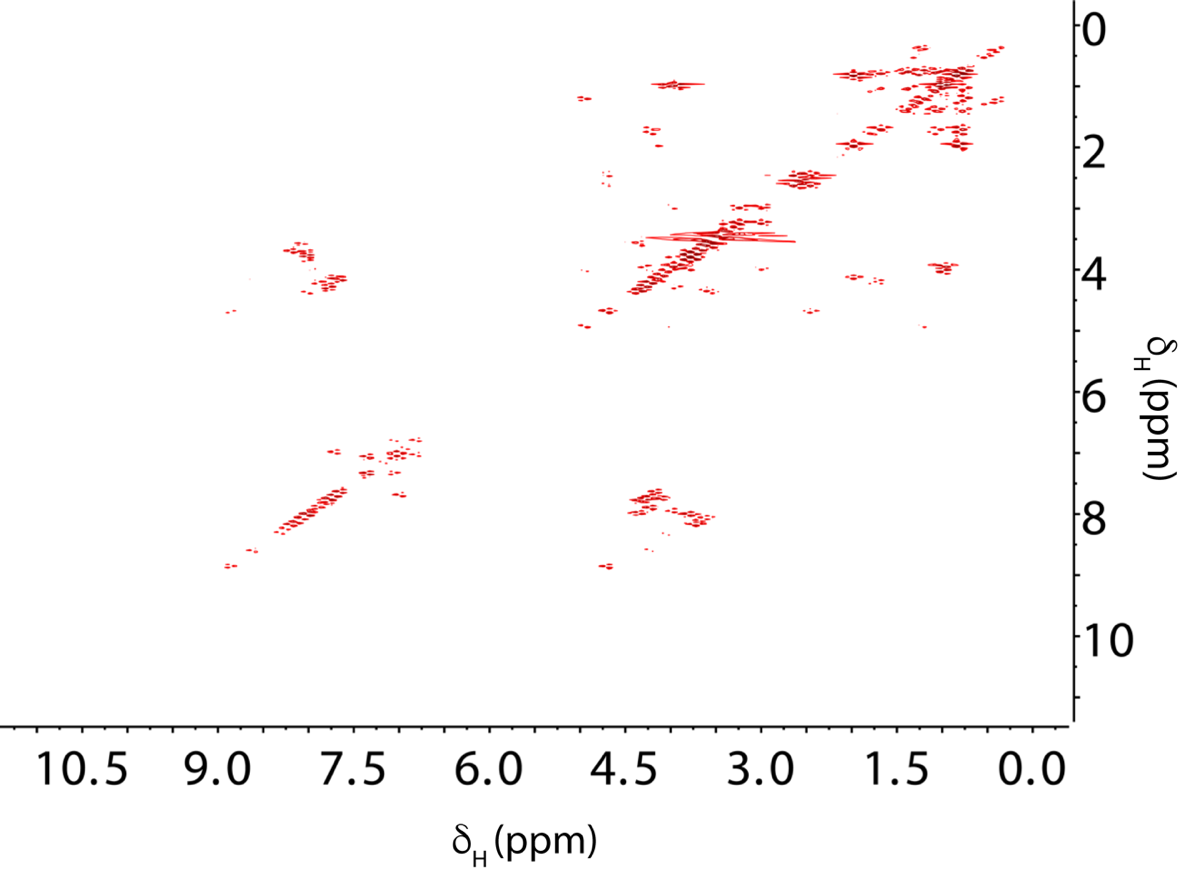


B)


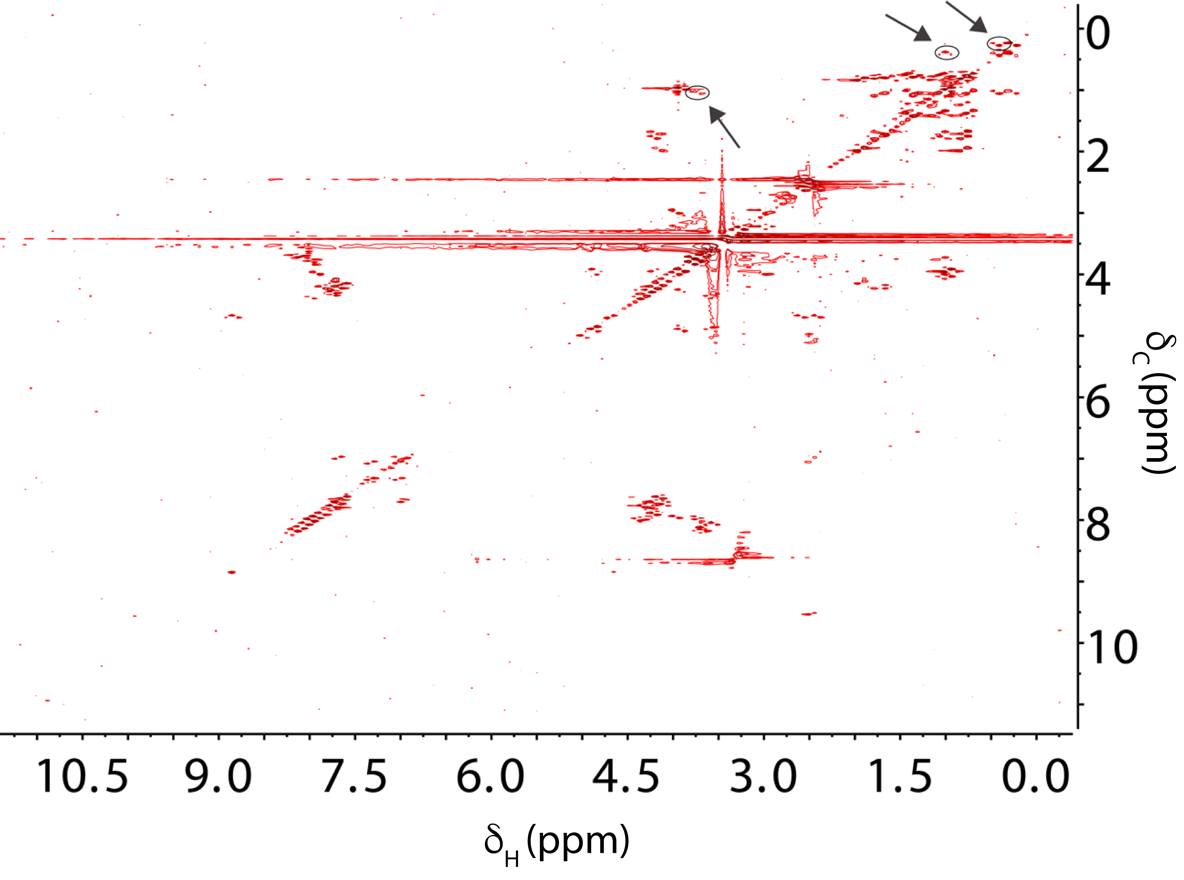


**Figure S9.** NMR spectra of the unmodified ^13^C_5_ ^15^N Val_4_ TvgA-4R variant in deuterated DMSO. Shown are **A)** ^1^H NMR; **B)** ^13^C NMR; **C)** ^13^C HSQC (recorded on Bruker Avance Neo 600 MHz with cold probe). **D)** ^13^C NMR; **E)** ^13^C CT-HSQC (recorded on Varian 900 MHz with cold probe).

A)


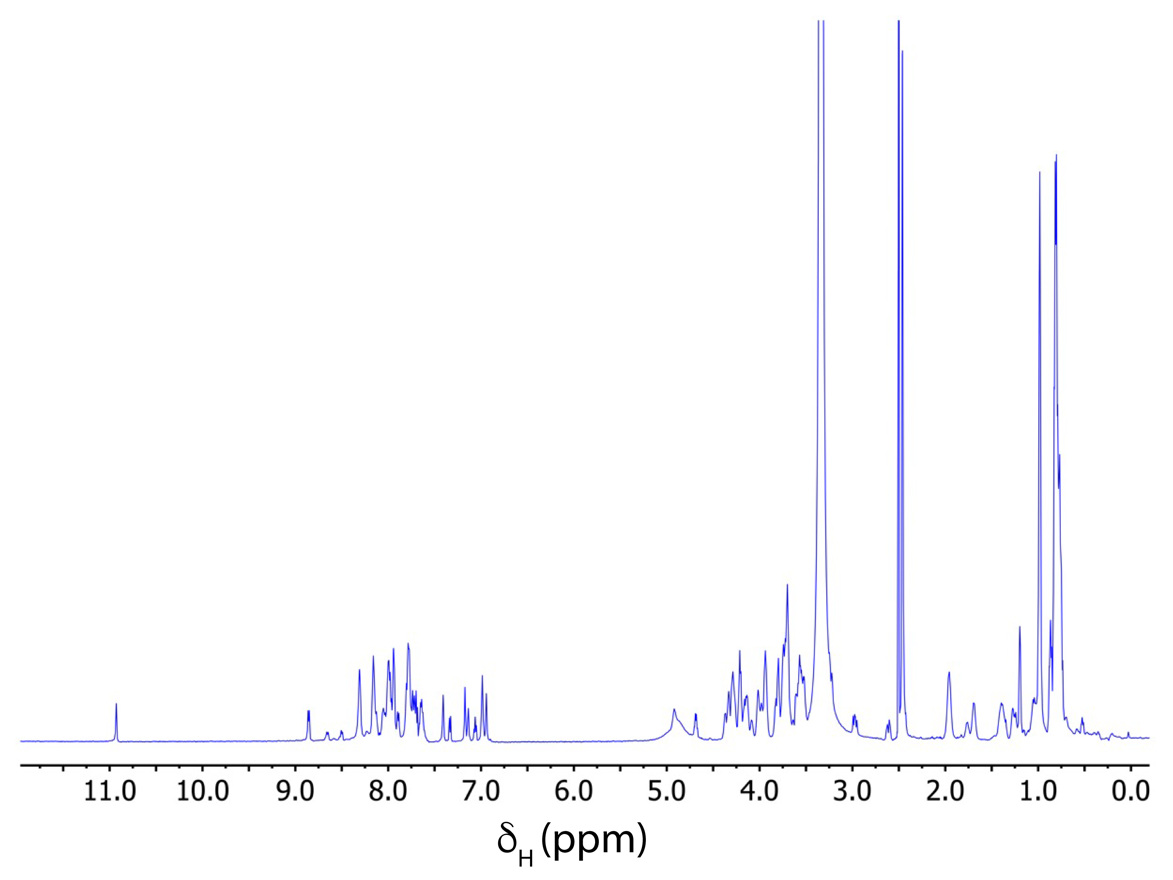


B)


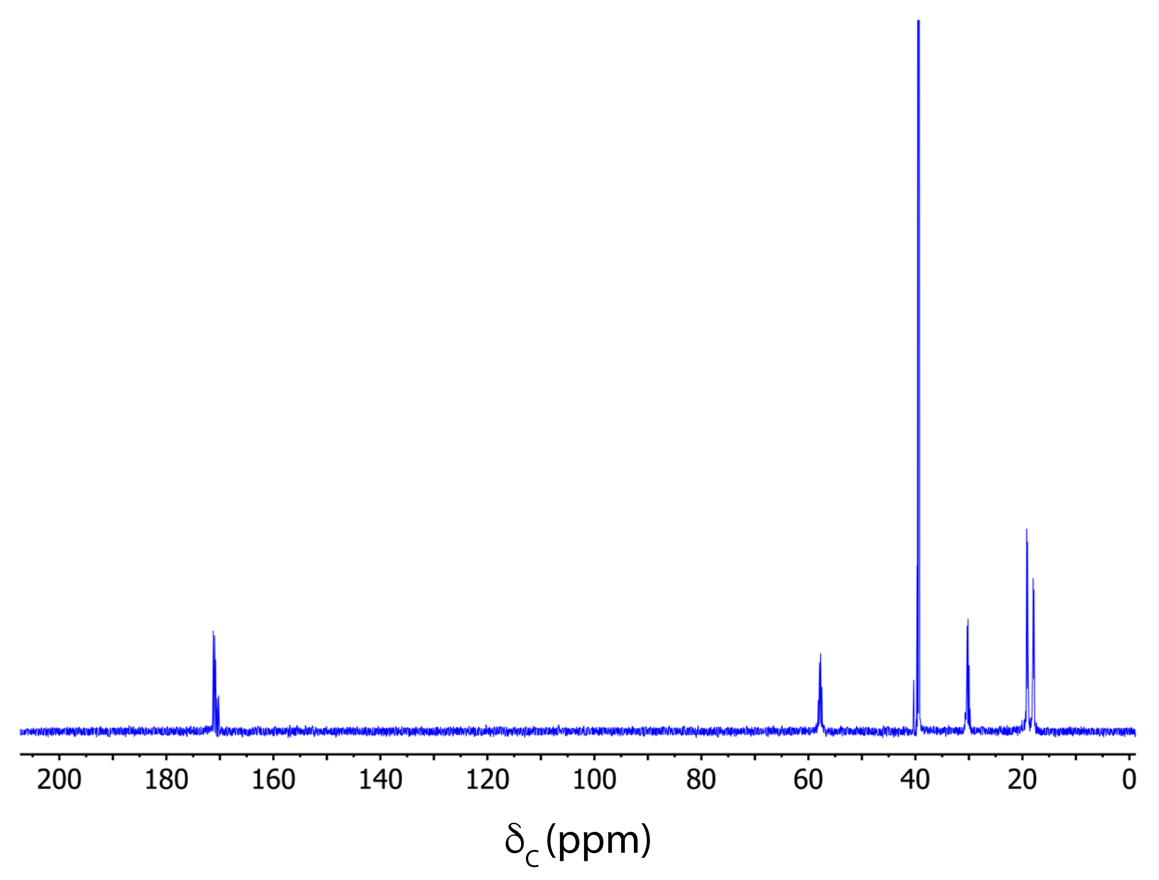


C)

**
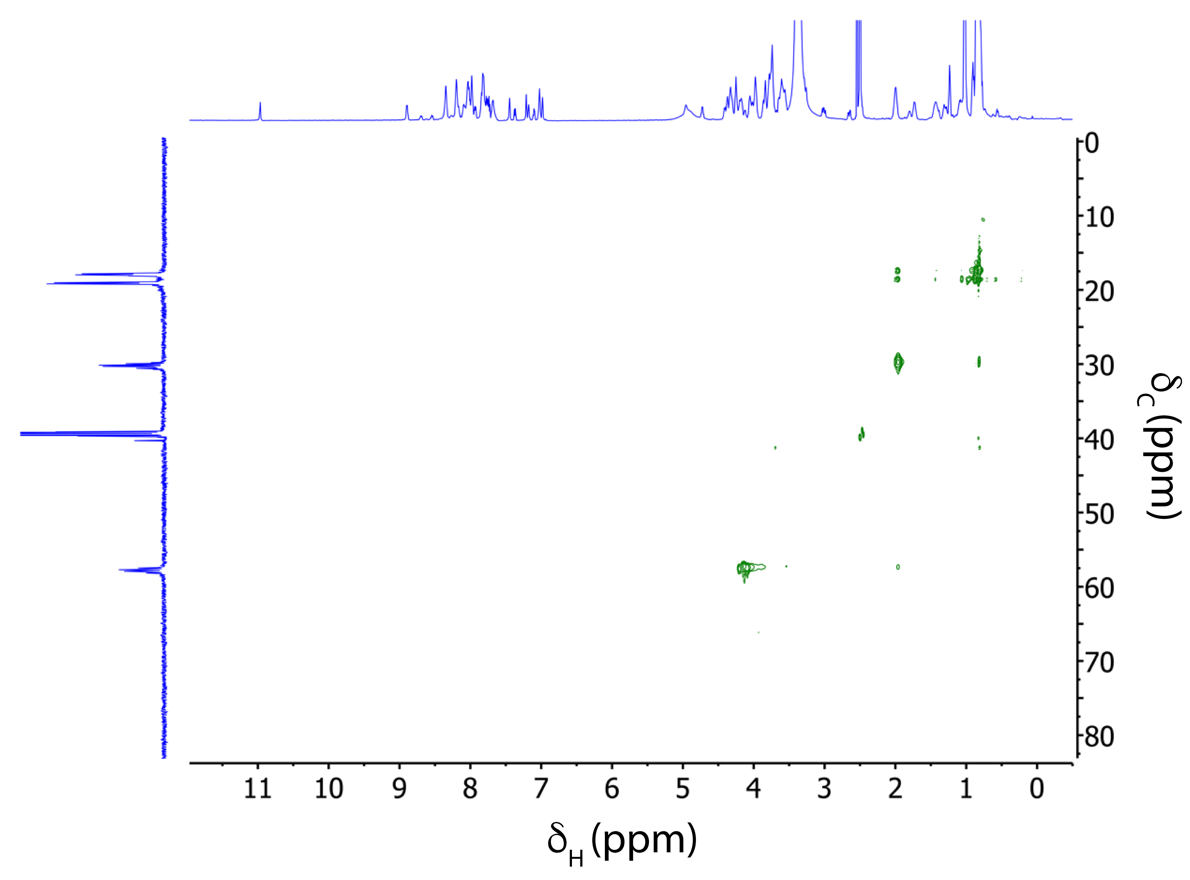
**

D)

**
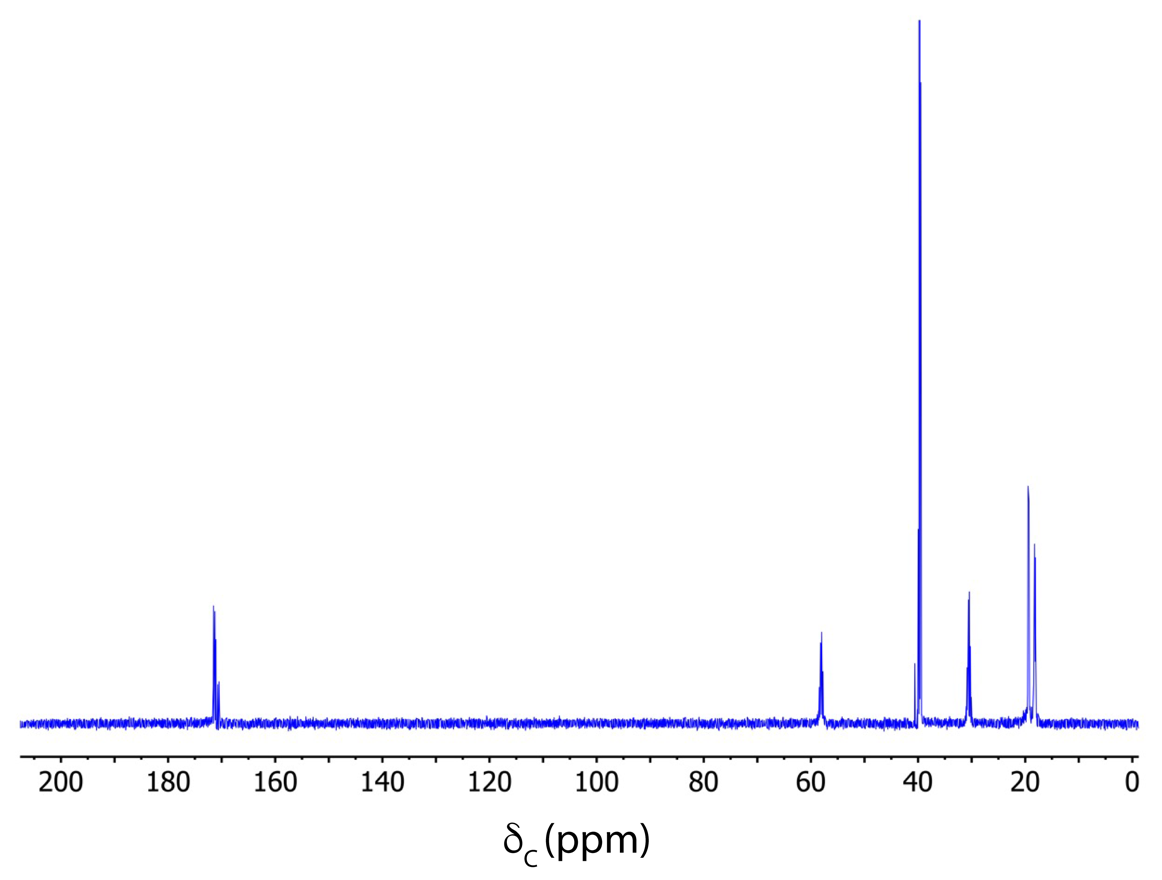
**

E)


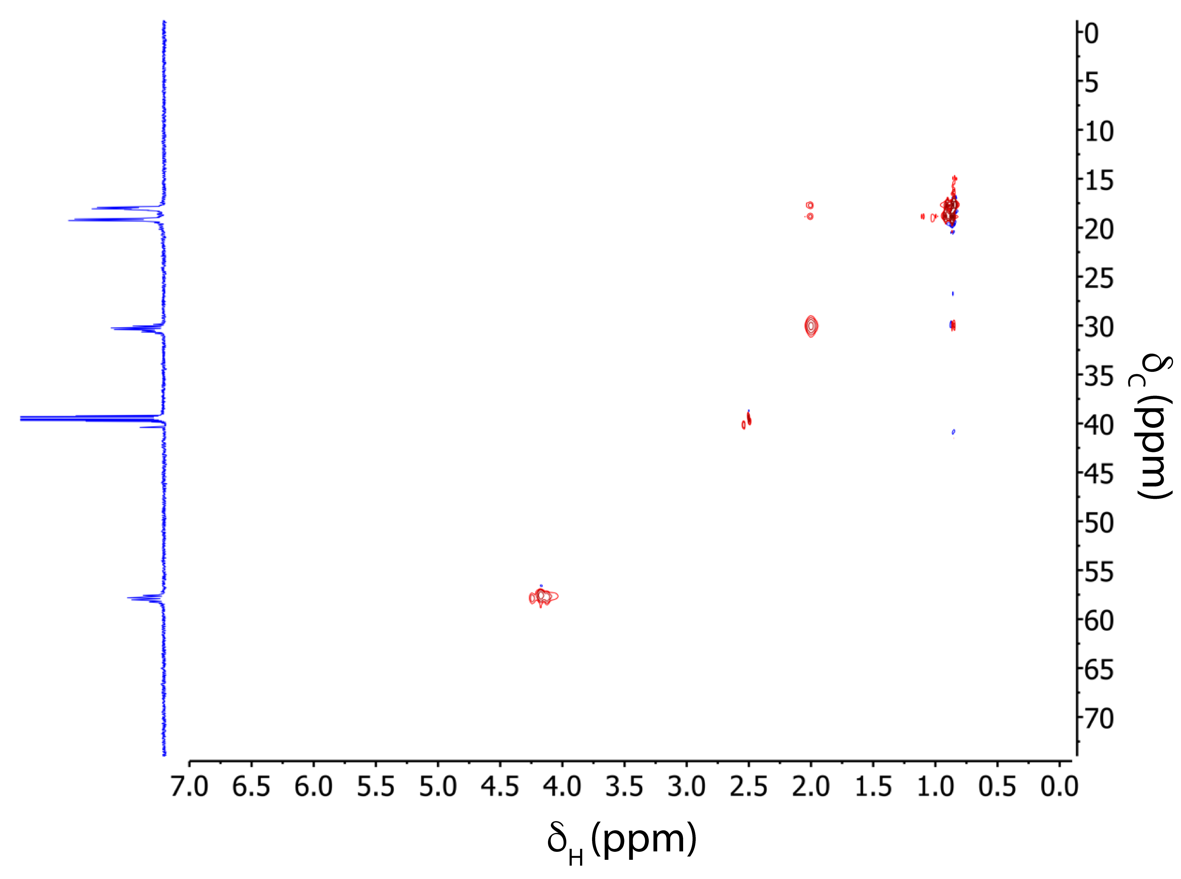


**Figure S10.** NMR spectra of the modified ^13^C_5_ ^15^N Val_4_ TvgA-4R variant in deuterated DMSO. Shown are **A)** ^1^H NMR; **B)** ^13^C NMR; **C)** ^13^C HSQC (recorded on Varian INOVA 600 MHz with cold probe). **D)** ^13^C NMR; **E)** ^13^C CT-HSQC (recorded on Varian 900 MHz with cold probe).

A)


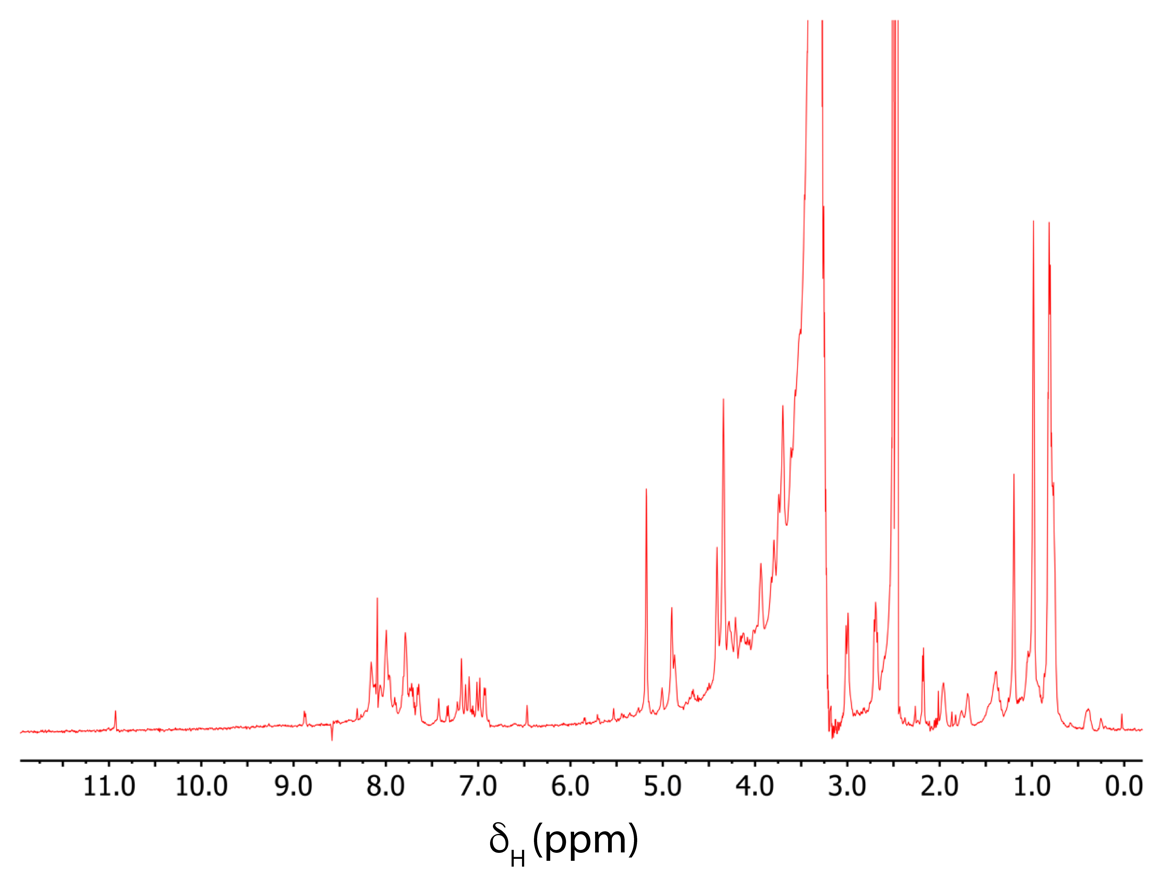


B)


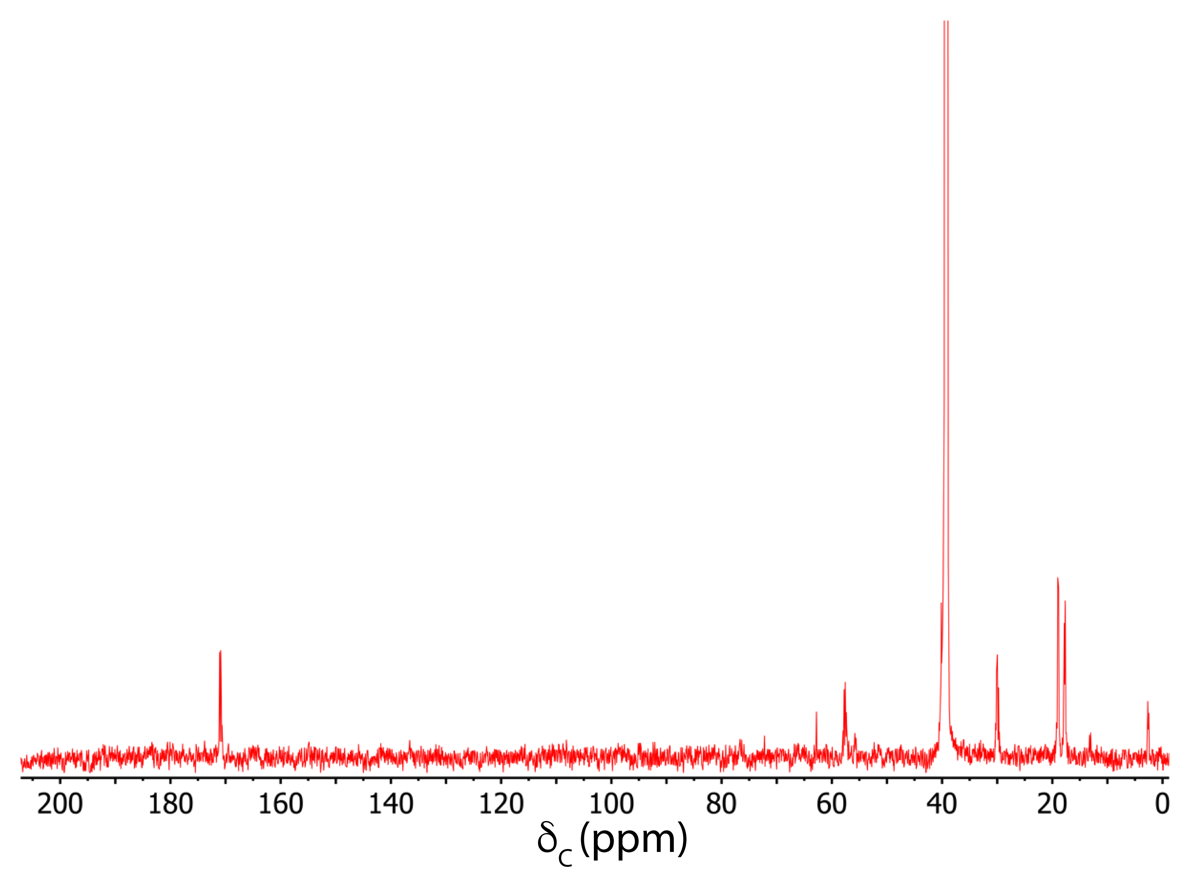


C)


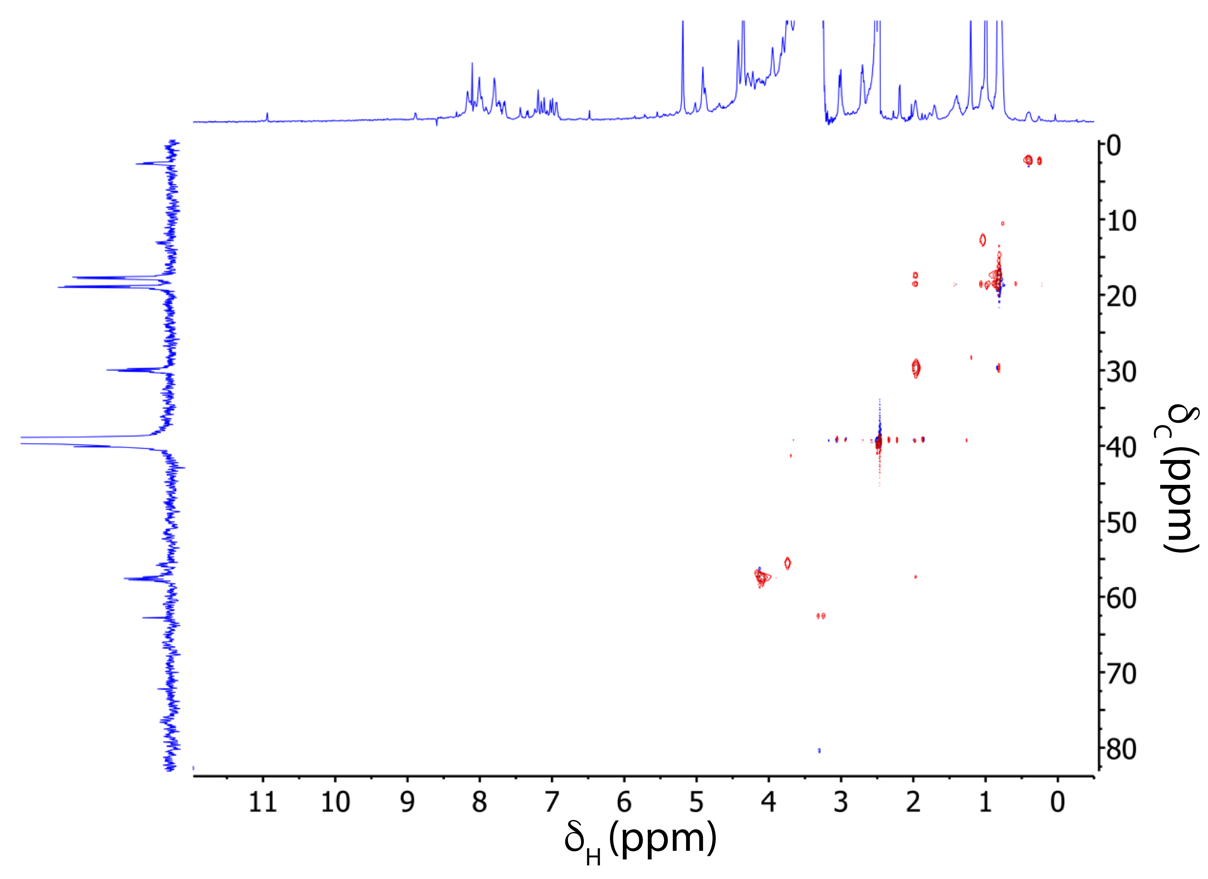


D)


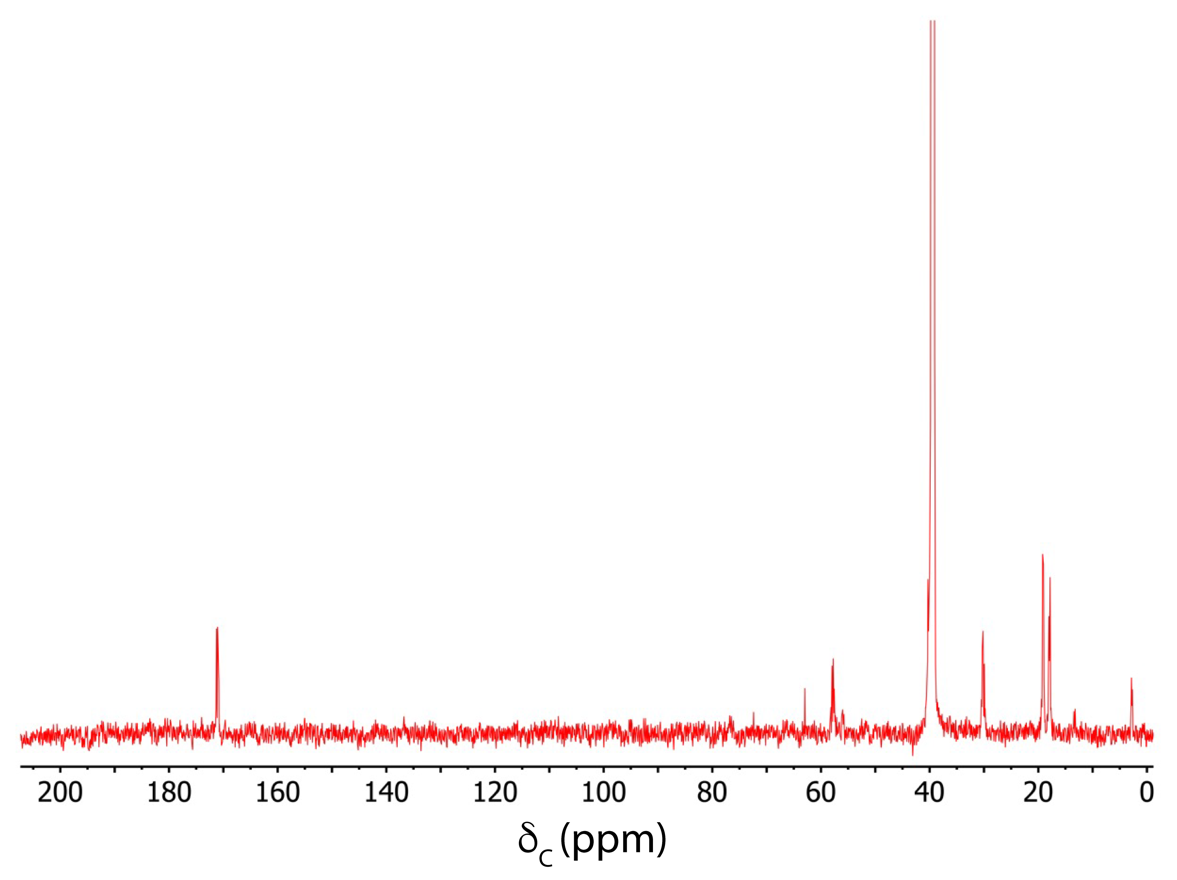


E)


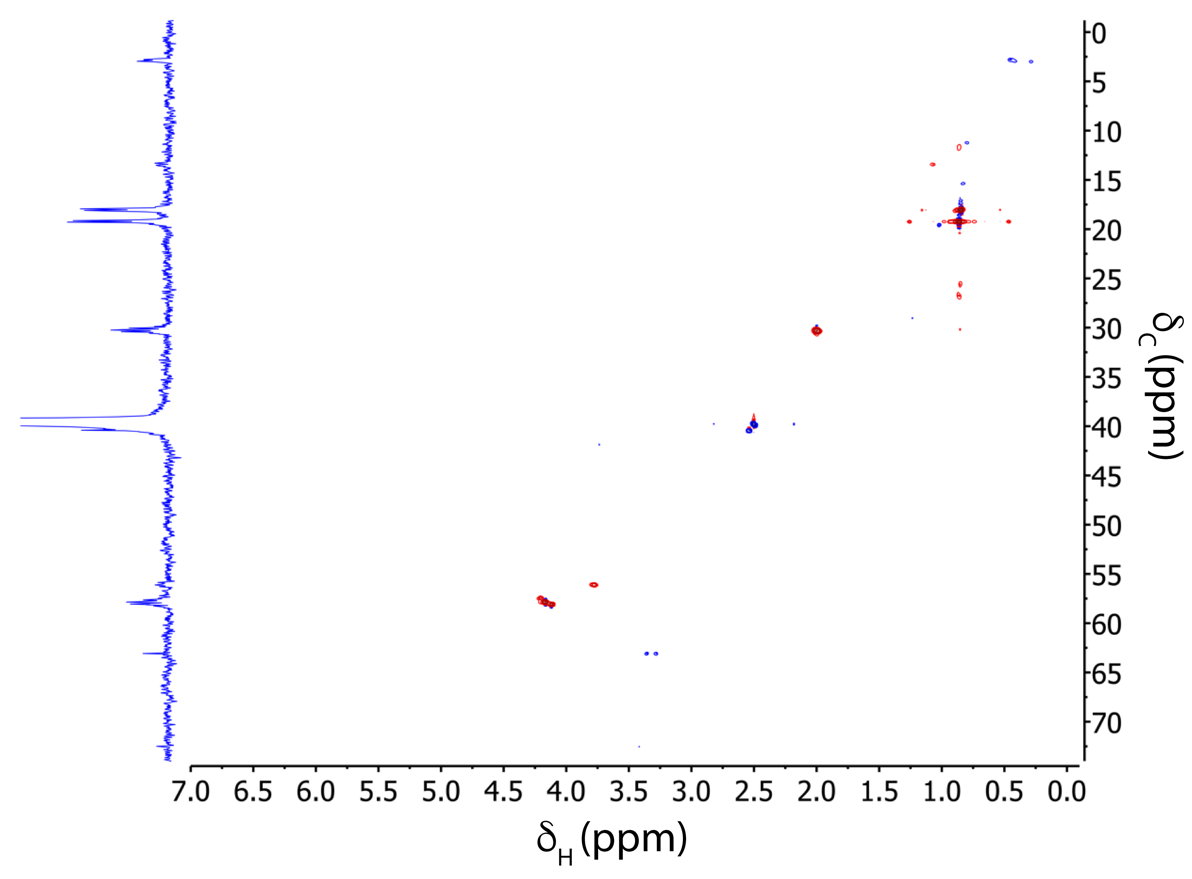


**Figure S11. A)** Overlayed ^13^C HSQC of TvgA-4R before (*green*) and after (*red*) the reaction with TvgB recorded in deuterated DMSO on Bruker 500 MHz. **B)** Stacked ^13^C NMR spectra of the unmodified (*blue*) and modified (*red*) ^13^C_5_ ^15^N Val_4_ TvgA-4R variant (recorded on Varian 900 MHz with cold probe). **C)** Overlayed ^13^C HSQC NMR spectra of the unmodified (*green*) and modified (*red*) ^13^C_5_ ^15^N Val_4_ TvgA-4R variant (recorded on Bruker Avance Neo 600 MHz with cold probe).

A)


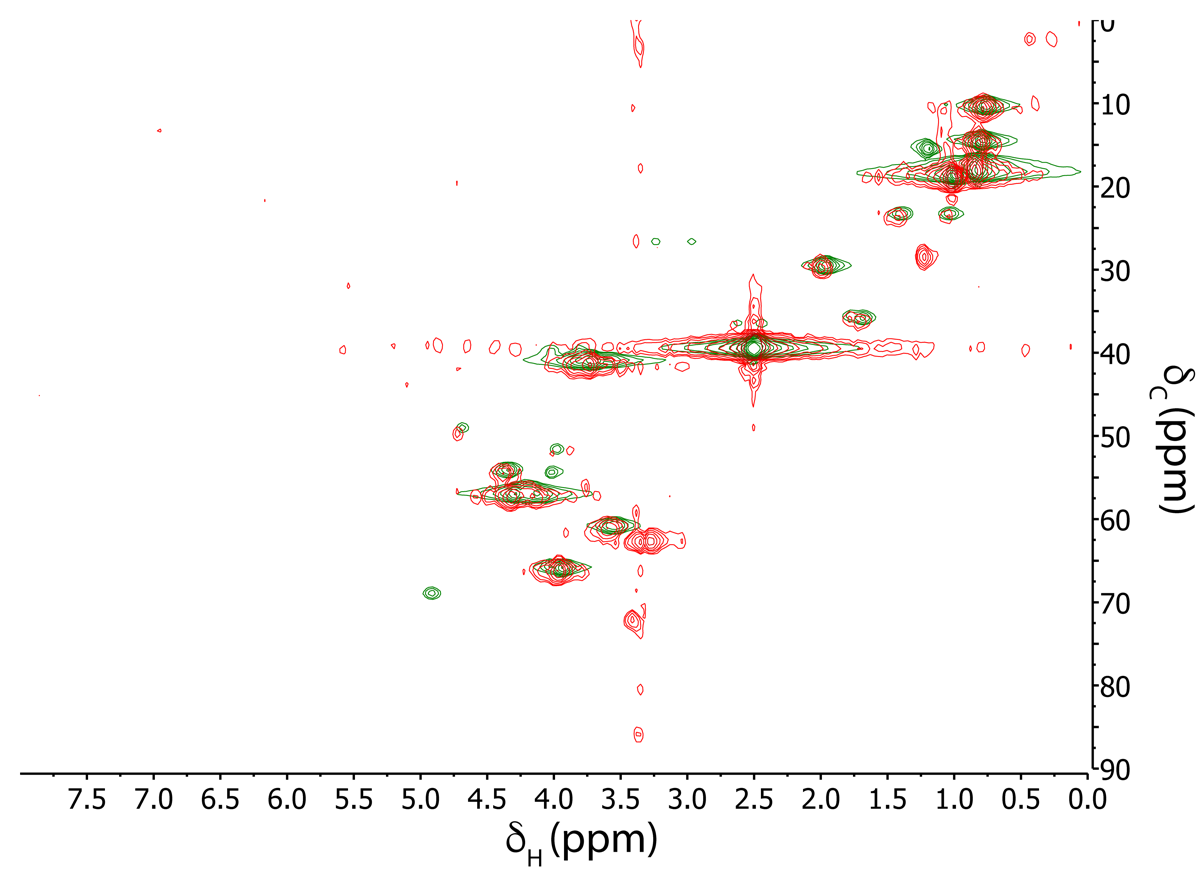


B)


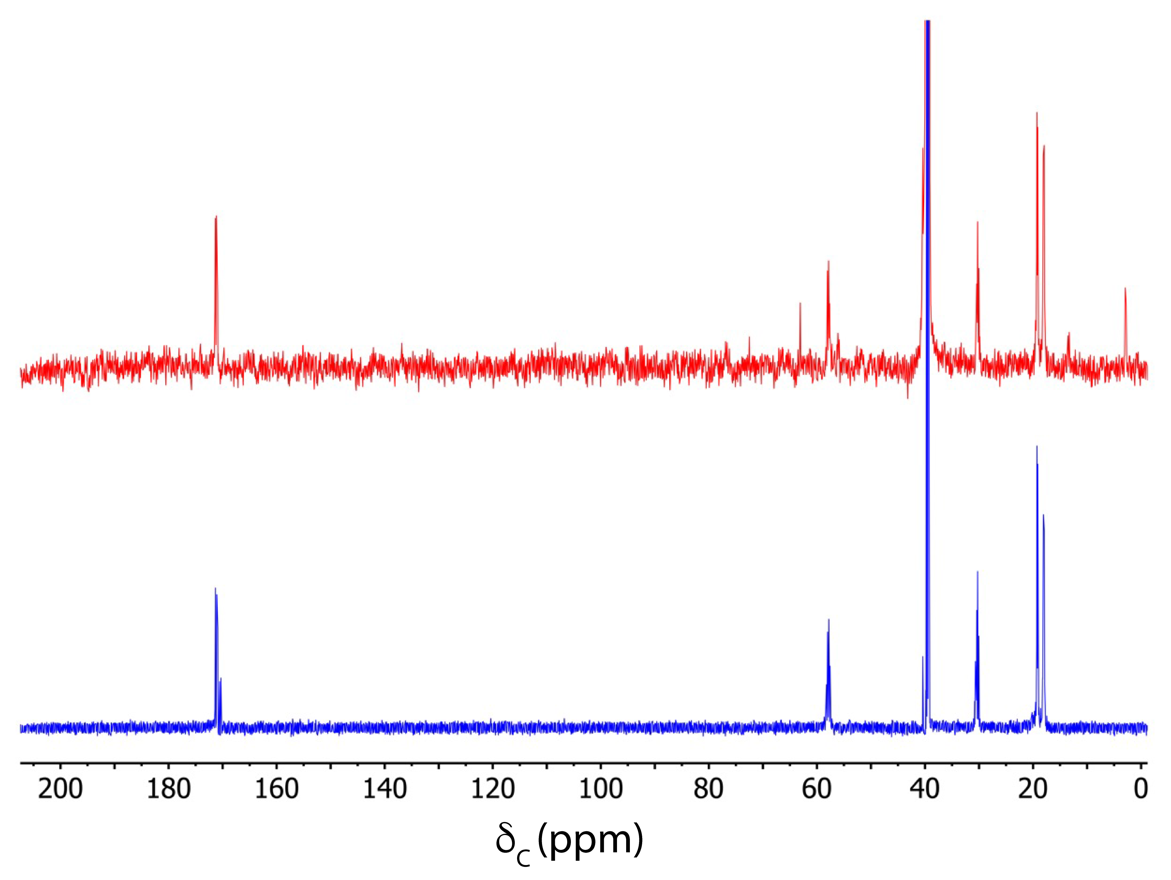


C)


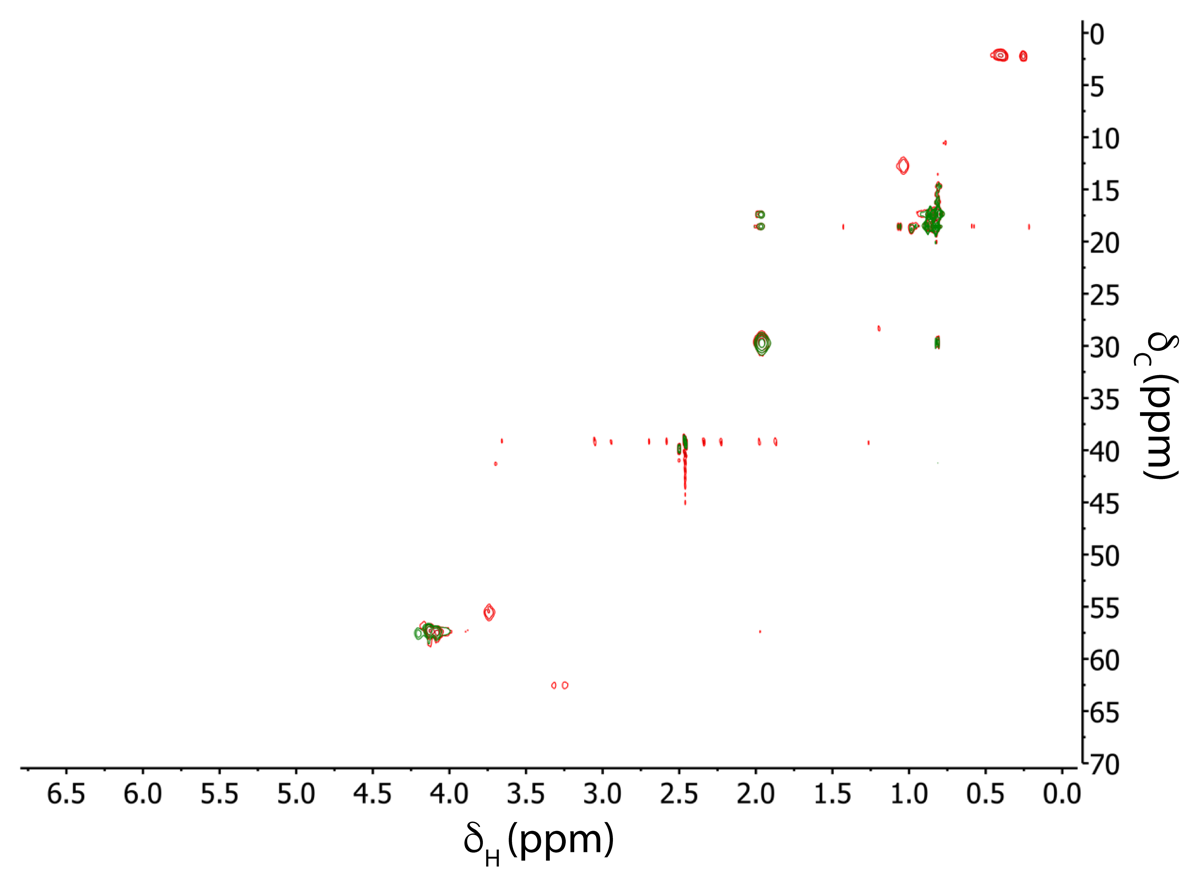


**Table S6.** ^1^H and ^13^C experimental chemical shifts for 13C Val TvgA-4R in DMSO-D_6_ and calculated chemical shifts for the model compound 1-cyclopropyl-1-(methylamino)propan-2-one. Reported experimental values were recorded on Varian 900 MHz hence the chemical shifts might differ from the ones recorded on Bruker Avance Neo 600 MHz.

| **δH** | | | **δC** | | |
| --- | --- | --- | --- | --- | --- |
| **C** | **Calculated** | **Observed** | **C** | **Calculated** | **Observed** |
| α | 3.35 | 3.78 | α | 59.4 | 55.95 |
| β | 1.47 | 1.08 | β | 12.1 | 13.34 |
| γ1 | 0.56/ 0.92 | not observed | γ1 | 5.1 | not observed |
| γ2 | 0.66/0.84 | 0.29/0.44 | γ2 | 3.0 | 2.84 |
|  |  |  | carbonyl | 171.4 | 171.05 |

**Figure S12.** ^13^C HSQC NMR spectra of the synthesized CPG_2_ TvgA-4R variant in deuterated DMSO. New signals observed in the modified TvgA-4R were also present in this spectrum, supporting the C-C bond formation between two methyl group of valine.


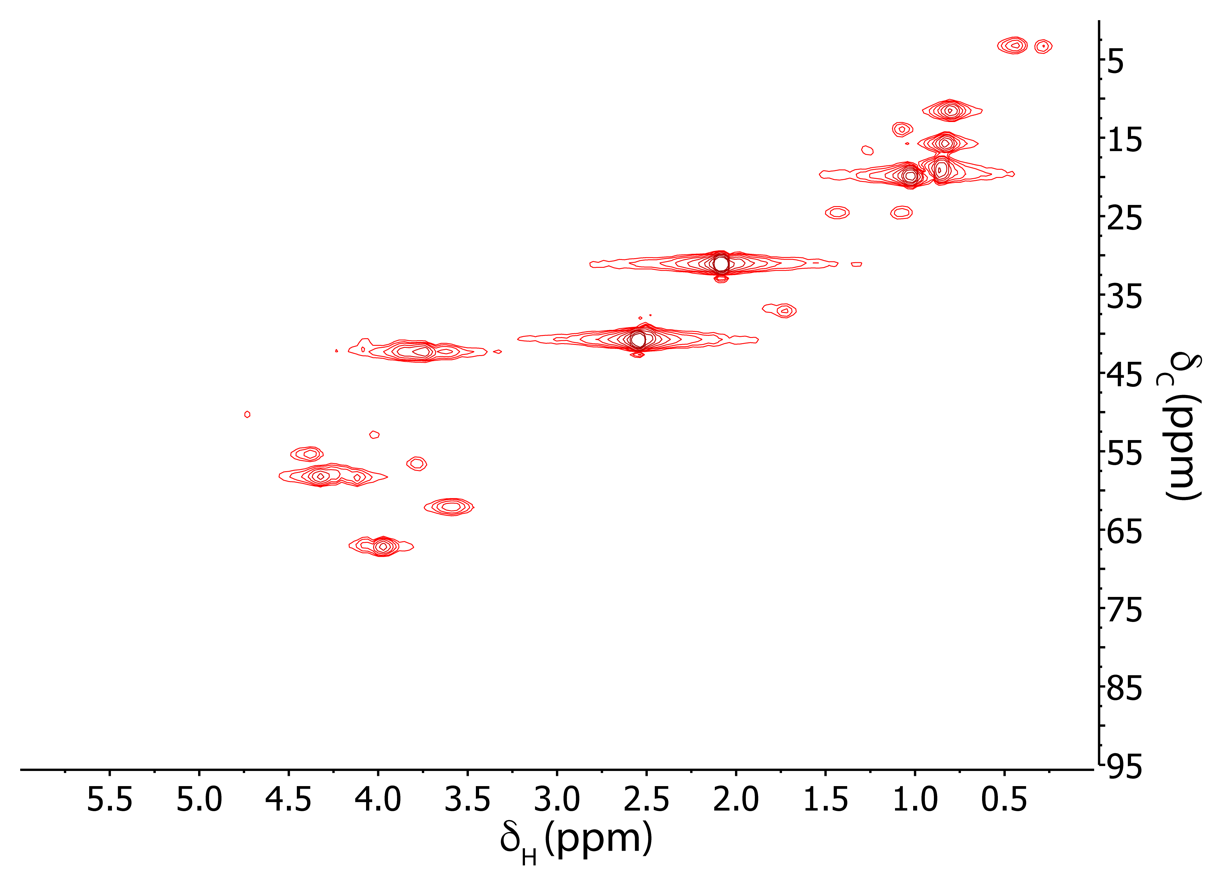


**Figure S13.** ^13^C HSQC NMR spectra of the modified ^13^C_4_ ^15^N Thr_4_ TvgA-4R variant in deuterated DMSO. No new signals were observed in the spectra eliminating participation of Thr in modification.


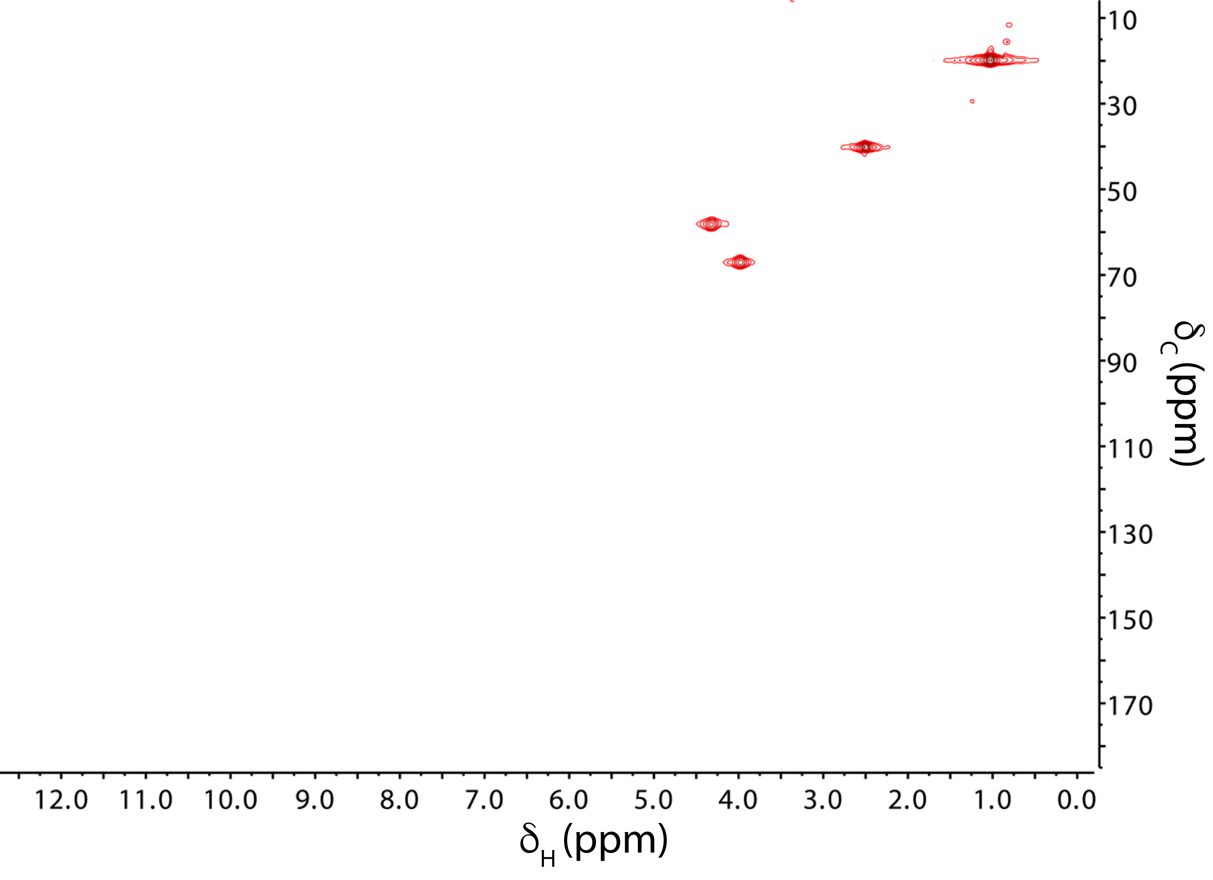

Supplement: Supplemental Figures S1–S13 and Tables S1–S6 [file mmc2.docx]
